# Predicting treatment resistance from first-episode psychosis using routinely collected clinical information

**DOI:** 10.1038/s44220-022-00001-z

**Published:** 2023-01-19

**Authors:** Emanuele F. Osimo, Benjamin I. Perry, Pavan Mallikarjun, Megan Pritchard, Jonathan Lewis, Asia Katunda, Graham K. Murray, Jesus Perez, Peter B. Jones, Rudolf N. Cardinal, Oliver D. Howes, Rachel Upthegrove, Golam M. Khandaker

**Affiliations:** 1 Imperial College London Institute of Clinical Sciences and UKRI MRC London Institute of Medical Sciences, Hammersmith Hospital Campus, London, UK; 2 Department of Psychiatry, University of Cambridge, Cambridge, UK; 3 Cambridgeshire and Peterborough NHS Foundation Trust, Cambridge, UK; 4 South London and Maudsley NHS Foundation Trust, London, UK; 5 Institute for Mental Health and Centre for Human Brain Health, University of Birmingham, Birmingham, England; 6 Birmingham Early Intervention Service, Birmingham Women’s and Children’s NHS Foundation trust; 7 Norwich Medical School, University of East Anglia. Norwich, UK; 8 Applied Research Collaboration East of England, National Institute for Health Research (NIHR), UK; 9 Institute of Biomedical Research of Salamanca (IBSAL); Psychiatry Unit, Department of Medicine, University of Salamanca, Salamanca, Spain; 10 Department of Psychosis Studies, Institute of Psychiatry, Psychology & Neuroscience, Kings College London, De Crespigny Park, London, SE5 8AF, UK; 11 MRC Integrative Epidemiology Unit, Population Health Sciences, Bristol Medical School, University of Bristol, Bristol, England; 12 Centre for Academic Mental Health, Population Health Sciences, Bristol Medical School, University of Bristol, Bristol, England

## Abstract

Around a quarter of people who experience a first episode of psychosis (FEP) will develop treatment-resistant schizophrenia (TRS), but there are currently no established clinically useful methods to predict this from baseline. We aimed to explore the predictive potential for clozapine use as a proxy for TRS of routinely collected, objective biomedical predictors at FEP onset, and to externally validate the model in a separate clinical sample of people with FEP. We developed and externally validated a forced-entry logistic regression risk prediction Model fOr cloZApine tReaTment, or MOZART, to predict up to 8-year risk of clozapine use from FEP using routinely recorded information including age, sex, ethnicity, triglycerides, alkaline phosphatase levels, and lymphocyte counts. We also produced a least-absolute shrinkage and selection operator (LASSO) based model, additionally including neutrophil count, smoking status, body mass index, and random glucose levels. The models were developed using data from two UK psychosis early intervention services (EIS) and externally validated in another UK EIS. Model performance was assessed via discrimination and calibration. We developed the models in 785 patients, and validated externally in 1,110 patients. Both models predicted clozapine use well at internal validation (MOZART: C 0.70; 95%CI 0.63,0.76; LASSO: 0.69; 95%CI 0.63,0.77). At external validation, discrimination performance reduced (MOZART: 0.63; 0.58,0.69; LASSO: 0.64; 0.58,0.69) but recovered after re-estimation of the lymphocyte predictor (C: 0.67; 0.62,0.73). Calibration plots showed good agreement between observed and predicted risk in the forced-entry model. We also present a decision-curve analysis and an online data visualisation tool. The use of routinely collected clinical information including blood-based biomarkers taken at FEP onset can help to predict the individual risk of clozapine use, and should be considered equally alongside other potentially useful information such as symptom scores in large-scale efforts to predict psychiatric outcomes.

## Introduction

Schizophrenia spectrum disorders can have remarkably different life courses: approximately half of people presenting with a first episode of psychosis (FEP) show good outcomes, such as remission^1^ or no need for long-term secondary care^2^. However, ~23-24% of FEP patients go on to develop treatment-resistant schizophrenia (TRS)^3^. TRS is typically defined as resistance to two antipsychotic treatments, each given at an adequate dose for at least 6 weeks, with evidence of medication adherence^4^. TRS is associated with reduced quality of life, substantial societal burden, and up to tenfold higher healthcare costs^5^.

It is not currently possible to predict accurately whether someone with FEP will develop TRS. This is important because there is evidence that clozapine, the only treatment licensed for TRS^6^, is more effective the sooner it is prescribed^7^. Yet, in clinical practice there are often long delays before clozapine is considered^8^. This highlights the need to identify treatment resistance as soon as possible.

Risk prediction in psychosis is a flourishing field, with the number of papers on the topic doubling between 2012 and 2019 (Supplementary Figure 1). However, in many existing studies the focus has been on trying to elucidate the pathophysiological underpinnings of treatment resistance, rather than the production of a clinically useful tool. While the former is an important research goal, it is distinct from the latter, which is of greater immediate clinical relevance. For example, existing studies have commonly included predictors that are, currently, either: not easy to deploy in routine clinical practice (e.g., neuroimaging^9^ or genetic measures^10^); not routinely or reliably collected (e.g., duration of untreated psychosis^11^, substancemisuse^12, 13^, premorbid functioning^14^); not available at FEP onset (e.g., antipsychotic medication polypharmacy during follow-up^15^, symptom patterns over time^12, 15^). Furthermore, some of the research has focussed on short term outcomes, such as clozapine use at the end of a current admission^16^. All these characteristics limit the potential clinical usefulness of existing efforts in TRS prediction.

Several studies have also attempted to combine variables to predict TRS or proxies (such as clozapine use), including diagnosis, symptom patterns, age at onset, genomic data, duration of untreated psychosis, and others^17^. A recent meta-analysis reported that, in addition to limited clinical usefulness, most previous studies are limited by methodological difficulties or poor reporting practices, particularly a lack of assessment of model calibration; a lack of external validation to assess generalizability^18, 19^, limited consideration of sample size and the risk of overfitting, and the inclusion of variables that cannot be known at FEP onset, such as medication during follow-up. While these limitations are by no means specific to TRS prediction studies^20, 21^, there is a clear need for studies that follow methodological best practices.

Blood biomarkers are commonly used to predict clinical outcomes in large-scale routinely used general population based risk prediction algorithms^22^. Blood biomarkers are objective, precise, and have advantages over self- or observer-rated questionnaires or interviews because they are not affected by inter-rater variability, recallor other biases. Indeed,biomarkers and clinical measures commonly taken at FEP onset can help predict clinical outcomes such as the development of metabolic syndrome in patients with psychosis^23^. Furthermore, meta-analyses of cross-sectional studies show that inflammatory and metabolic alterations are already evident in antipsychotic-naïve patients with FEP, including impaired glucose tolerance, insulin resistance^24^, hypertriglyceridemia^25^, and pro-inflammatory changes^26^. These biomarkers may be associated with a more chronic psychiatric illness course^2, 27^. Furthermore, elevated lipid levels may predate the development of non-alcoholic fatty liver disease (NAFLD)^28^, which is associated with schizophrenia^29^.

In this work, we aimed to use routinely collected, objective and measurable biomedical predictors at FEP onset to predict clozapine use (as a proxy for TRS) up to 8 years later, with the aim of producing the most parsimonious prediction model with the potential for clinical use. This work focusses on the pragmatic, operational definition of both predictors and outcomes, to foster greater confidence in their validity and to allow easy replicability world-wide. We used patient data from three UK early intervention psychosis services (EISs) to investigate the predictive potential of sociodemographic, lifestyle, and biological data routinely recorded at FEP baseline. We aimed to follow methodological and reporting best practices, for example by including an external validation step to examine generalizability and thus potential usefulness. We performed sensitivity analyses to examine the incremental improvement in prediction attributable to different measures, and followed the Transparent Reporting of a Multivariable Prediction Model for Individual Prognosis or Diagnosis (TRIPOD) guidelines (see Supplementary Table 1).

## Methods and Materials

### Data sources

#### Model Development Sample

We developed a risk prediction model using pooled longitudinal data from patients enrolled in the Cambridgeshire and Peterborough Assessing, Managing and Enhancing Outcomes (CAMEO) psychosis EIS (sampling frame n=1,660) or the Birmingham EIS (sampling frame n=391). This was selected as the development sample for the present study as CAMEO data were recently used to examine group-level associations between mean biomarker levels and psychiatric outcomes^2^.

Predictors were assessed within 100 days of patient EIS enrolment. We excluded any participant who had missing data on >50% predictor variables, and non-cases (patients who did not use clozapine) who had less than 2 years of follow-up to reduce the probability of including future TRS cases as non-cases. All patients who developed TRS were included regardless of duration of follow-up. As predictors must pre-date outcomes, we also excluded all cases where the outcome start date (clozapine treatment start date, see below) pre-dated the earliest available baseline bloods in the CAMEO cohort (and SLaM cohort, see below), or participants who started taking clozapine within 100 days of baseline in the Birmingham cohort. Please see the Supplementary Methods for further information on the development sample.

#### Model External Validation Sample

We used the Clinical Records Interactive Search (CRIS) resource to capture anonymised data from South London and Maudsley NHS Foundation Trust (SLaM) EIS (National Institute for Health Research [NIHR] Biomedical Research Centre [BRC] CRIS Oversight Committee reference 20-005). Our sampling frame included 3,012 EIS patients, all those enrolled between 2012-01-01 and 2021-11-20. Patients were excluded and predictors and outcomes were assessed as for the development sample.

### Outcome

Due to data availability, we adopted a pragmatic definition of TRS: patients were defined as having TRS if they had been treated with clozapine at any point during the follow-up period. Clozapine is the only clinically approved treatment for TRS in the UK, and provides an objective, easily quantifiable measure of TRS^30^. We calculated an expected prevalence of clozapine use of 13%. This was calculated as follows: starting from a population prevalence of 23%^3, 14, 31^, we expected to capture mostly "early onset" cases, which represent ~84% of cases^11^. From previous literature, clozapine is given in ~68% of TRS cases^11^, so the expected prevalence was = 0.23 * 0.84 * 0.68 = 0.13.

### Predictor variables

Routinely used clinical predictors were included based on a balance of clinical knowledge, existing research, and likely clinical usefulness. Demographic variables were considered if they had shown evidence of potential predictive ability for TRS in existing prognosis research^17, 18^. Biomarkers and clinical measures were considered if they showed evidence from past longitudinal association studies of biological measures at FEP using long-term clinical outcomes^2, 27^. Predictors were only included if they were part of the suite of measurements that should be collected at baseline as part of local or national guidelines, to avoid ascertainment bias. We did not include variables that may only be recorded in specific circumstances, such as C-reactive protein, which may only be recorded when an infection is suspected. All predictors needed to be available in all three EIS samples. Therefore, we considered the following parameters, measured within 100 days of EIS start: sex (female or male); age (years); ethnicity (categorical: white European or not recorded [reference], Black or African-Caribbean, Asian, or other); triglyceride concentration (mmol/L); lymphocyte and neutrophil blood cell counts (billion/L); alkaline phosphatase levels (ALP, units/L), smoking status (binary, at least one cigarette on average daily); body mass index (BMI, kg/m^2^); and random glucose levels (mmol/L).

See Supplementary Methods for full rationale and details of data extraction.

### Statistical analysis

#### Primary Analysis

We performed sample size calculations using the R package *pmsampsize*^32^. The sample size required was estimated from the estimated outcome prevalence, the *a priori* estimated R^2^ of the model, and the estimated required model shrinkage. For 11 predictors, the minimum sample required was 412. We did not consider non-linear terms or interactions to reduce the risk of overfitting. See Supplementary Methods for detailed sample size calculations.

We used multiple imputation using chained equations for missing data, and pooled estimates using Rubin’s rules (see Supplementary Methods for details about predictor missingness). Internal validation involved bootstrap resampling (500 bootstraps) to obtain an estimate of the corrected calibration slope. The resulting pooled corrected C slopewas then used as a shrinkage factor for our coefficients. After this step, predictive performance was assessed (see below).

We developed the risk calculator using two alternative model selection methods:

1. A forced-entry logisticregression model, including all sociodemographic and three biological predictors (one lipid, one inflammatory, and one liver marker), based on a balance of clinical knowledge, past research, and likely clinical usefulness (see above).
2. A least absolute shrinkage and selection operator (LASSO)-based selection model, after predictor scaling and centering, including all 11 pre-selected sociodemographic, lifestyle and biological predictors. The inclusion of additional variables was enabled by LASSO including a predictor selection step, and by its more efficient coefficient shrinkage, leading to less risk of model overfit^33^. For the LASSO model we used 100-fold cross-validation to tune the penalty parameter in the development sample as implemented in glmnet^34^.

Both methods involved variable pre-selection, after ruling out predictor multi-collinearity to minimise risk of overfitting, as is recommended for smaller datasets^35^.

The models were applied to the external validation sample. The distribution of predicted outcome probabilities was inspected using histograms.

Model performance was assessed primarily with measures of *discrimination* (the ability of the model to distinguish participants with the outcome from those without), such as the C statistic, and *calibration* (the extent to which the outcome probabilities predicted by the model in specified risk-defined subgroups are similar to those observed in the validation dataset), assessed by inspection of calibration plots (presented as figures).

The discrimination of the models was assessed using the concordance (C) statistic; for binary outcomes this is equivalent to the area under the receiver operating characteristic (ROC) curve^35^, which plots sensitivity against 1 minus specificity. The C-statistic normally ranges from .5 to 1, with a value of 1 representing perfect discrimination and a value of .5 representing discrimination no better than chance. C-statistics were determined in relation to the observed binary outcomes (subsequent clozapine use or not).

We also recorded calibration intercepts (ideally close to 0) and Brier scores (an overall measure of model performance, ideally close to 0, with scores >0.25 generally indicating a poor model). For further details of our prediction methods, see^23^.

#### Model recalibration

Additionally, where performance at external validation differed from internal validation performance, we considered two recalibration approaches. First, we considered logistic recalibration. This method is used where the coefficients of the original model may have been over-fitted, affecting calibration performance. Logistic recalibration assumes similar relative effects of the predictors, but allows for a larger or smaller absolute effect of the predictors^36^. Further details are in Supplementary Methods. Second, where there was evidence of a clear difference in the association of a predictor with clozapine use between the development and validation samples, we considered logistic recalibration *plus* revising a single predictor in the model. We limited this model revision approach to a maximum of one model predictor, to preserve as much of the character of an external validation analysis as possible, though we note that all recalibrated/revised models will require a further external validation in an additional unseen sample.

#### Decision Curve Analysis

Decision curve analysis was performed to assess potential clinical benefit^37^. Clinical net benefit of the prediction model is calculated against offering an intervention to all or no patients. This can be calculated at a range of propensity to intervene thresholds. Net benefit is defined as the minimum probability of clozapine use at which the intervention would be warranted, as net benefit = sensitivity × prevalence – (1 – specificity) × (1 – prevalence) × *w*, where *w* is the odds at the propensity to intervene threshold^38^.In decision curve analysis, it is usual to only consider the range of propensity to intervene thresholds that may be clinically relevant; these depend on how risky the intervention being offered might be.

For starting clozapine, we selected *a priori* apropensity to intervene threshold of 0.50,representing a >50% risk of developing TRS. We believe that such a threshold would represent a good balance between the potential positives of early clozapine initiation, and relatively rare risks of clozapine. We also selected a lower propensity to intervene threshold of 0.10(>10% risk of developing TRS) for defining a "TRS-at risk population" who may be eligible for close monitoring.

The decision curve plot is presented as a figure, to visualise the net benefit of both model versions (forced-entry original, and recalibrated) over varying propensity to intervene thresholds compared with treating all patients or no-one. Classical decision theory proposes that at a chosen propensity to intervene threshold, the choice with the greatest net benefit should be preferred^37^.

#### Sensitivity Analysis

To examine the added benefit of selected demographic and biological predictors, we examined iterative improvements of the model. The first model included only a single demographic predictor, sex; the second added all demographics; the third included all demographics plus a single biological predictor (triglycerides); the last model included all the above plus a second biological predictor (ALP). We did not externally validate the incremental models.

### Visual representation of the model

We developed an online data visualisation tool using *shiny* for R, allowing interactive exploration of the effect of sociodemographic, lifestyle, and clinical variables and their combinations on TRS risk in people with FEP. The tool is not yet suitable for clinical use.

## Results

### Model development

**Figure 1:**
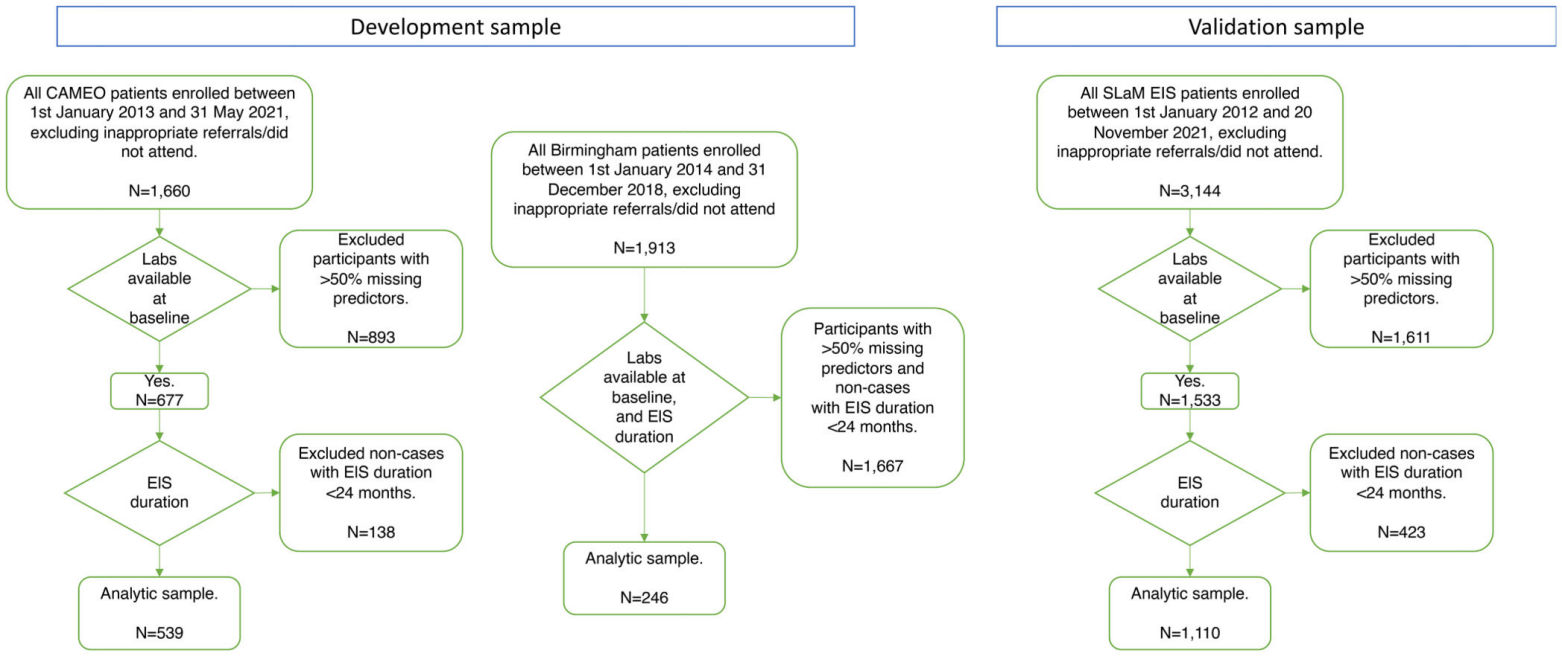
Patient selection flow-chart, by cohort

Data from 785 patients were included in the pooled development sample: 539 from CAMEO and 246 from the Birmingham EIS (Table 1), following EHR searches and application of inclusion and exclusion criteria (see flow-chart in Figure 1, and a description of the included and excluded samples in Supplementary Table 2).

Included patients were 28.2 years old, 66% white, 41% smokers, with an average BMI of 25. In the pooled development sample, 58 (7.4%) patients were treated with clozapine.

Model coefficients are presented in Table 2. Histograms of predicted outcome probabilities are provided as Supplementary Figures 2 and 3.

Univariable logistic regression coefficients (clozapine ~ predictor) are presented as Supplementary Table 3.

### Internal Validation

Measures of pooled internal validation performance of the models over 100 imputed datasets are shown in Table 2. The C statistic for the forced-entry model (MOZART) was 0.70 (95% confidence interval (CI): 0.63-0.76), while that for the LASSO model was 0.69 (95%CI: 0.63-0.77). Calibration plots showed good agreement between observed and expected risk at most predicted probabilities for both models, although the LASSO model showed slight overprediction of riskat lower predicted probabilities (Supplementary Figures 4 and 5).

### External validation

The external validation sample comprised 1,110 patients from the SLaM EIS (Table 1). Applying the models developed in the joint development sample to the SLaM EIS sample, the C statistic for MOZART was 0.63 (95% CI: 0.58-0.69), while that for the LASSO model was 0.64 (95%CI: 0.58-0.69) (Table 2).

**Figure 2:**
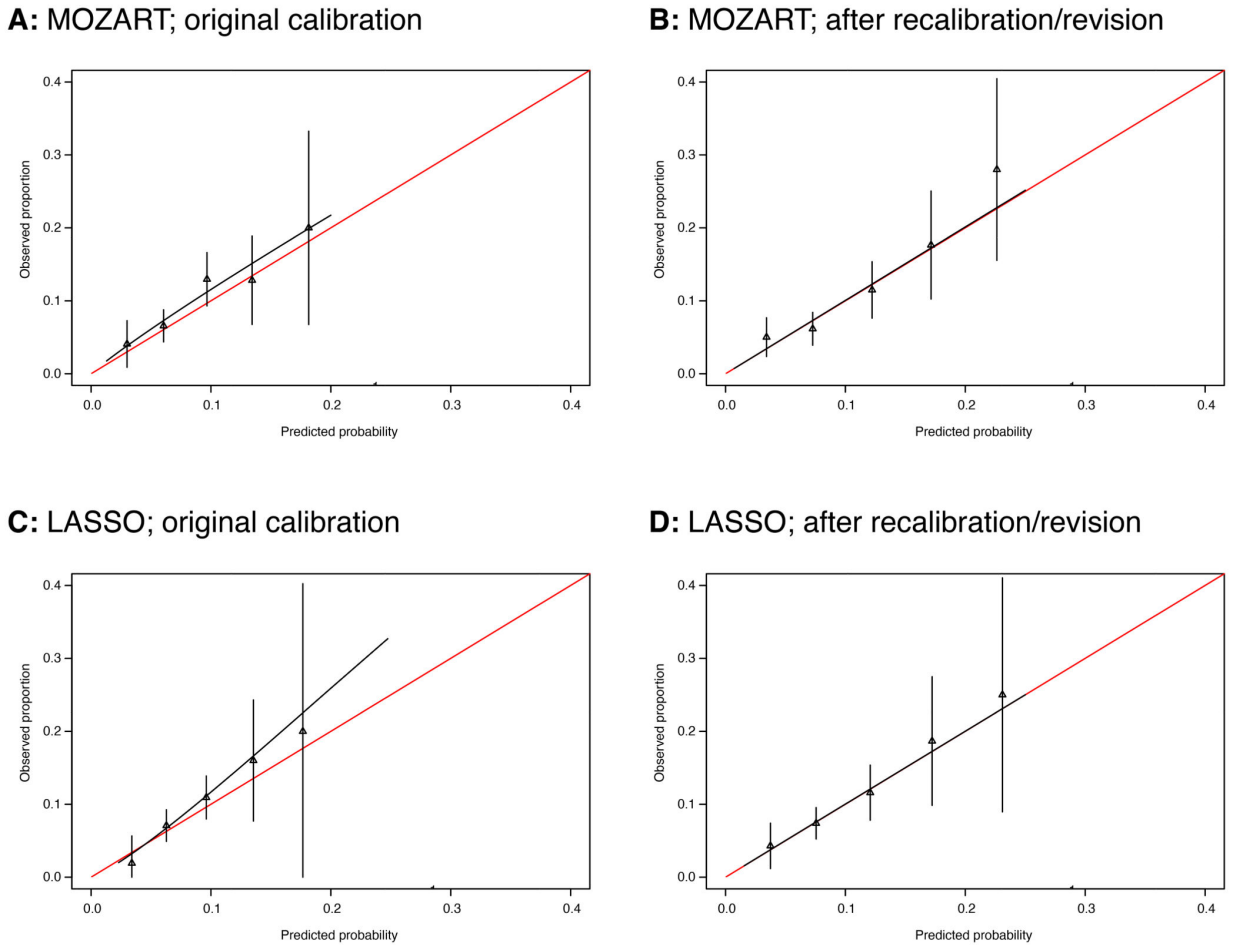
External validation calibration plots for the main models *Model calibration* is the extent to which outcomes predicted by the model are similar to those observed in the validation dataset. Calibration plots illustrate agreement between observed risk (y axis) and predicted risk (x axis). Perfect agreement would trace the red line. Model calibration is shown by the continuous black line. Triangles denote grouped observations for participants at deciles of predicted risk, with 95% CIs indicated by the vertical black lines. Axes range between 0 and 0.3 since very few individuals received predicted probabilities greater than 0.3. Panels A and B show external validation calibration plots for the forced-entry model (MOZART); A) shows calibration before, and B) shows calibration after recalibration. Panels C and D show external validation calibration plots for the LASSO model; C) shows calibration before, and D) shows calibration after recalibration.

The calibration plot for MOZART showed good agreement between observed and expected risk (Figure 2A), while that for the LASSO model showed evidence of mild overprediction of risk at higher predicted probabilities and of slight overprediction for very low risk (Figure 2C). In all models, the 95% CIs widened as predicted probabilities became higher, owing to lower numbers of participants.

### External validation after logistic re-calibration and model revision

We applied logistic recalibration to both main models in the external validation sample. The coefficient for lymphocyte count was selected for revision as the sign of the coefficient was reversed between the development and validation samples.

Table 2 shows that, after MOZART recalibration/revision, the C statistic was restored to values close to internal validation performance (C statistic = 0.67, 95% CI: 0.62-0.73). The same procedure performed on the LASSO model, however, did not produce any improvement on the original model performance statistics.

The calibration plots for both recalibrated models are shown in Figures 2B and 2D. Both showed good agreement between observed and expected risk.

### Decision curve analysis and data visualisation tool

**Figure 3:**
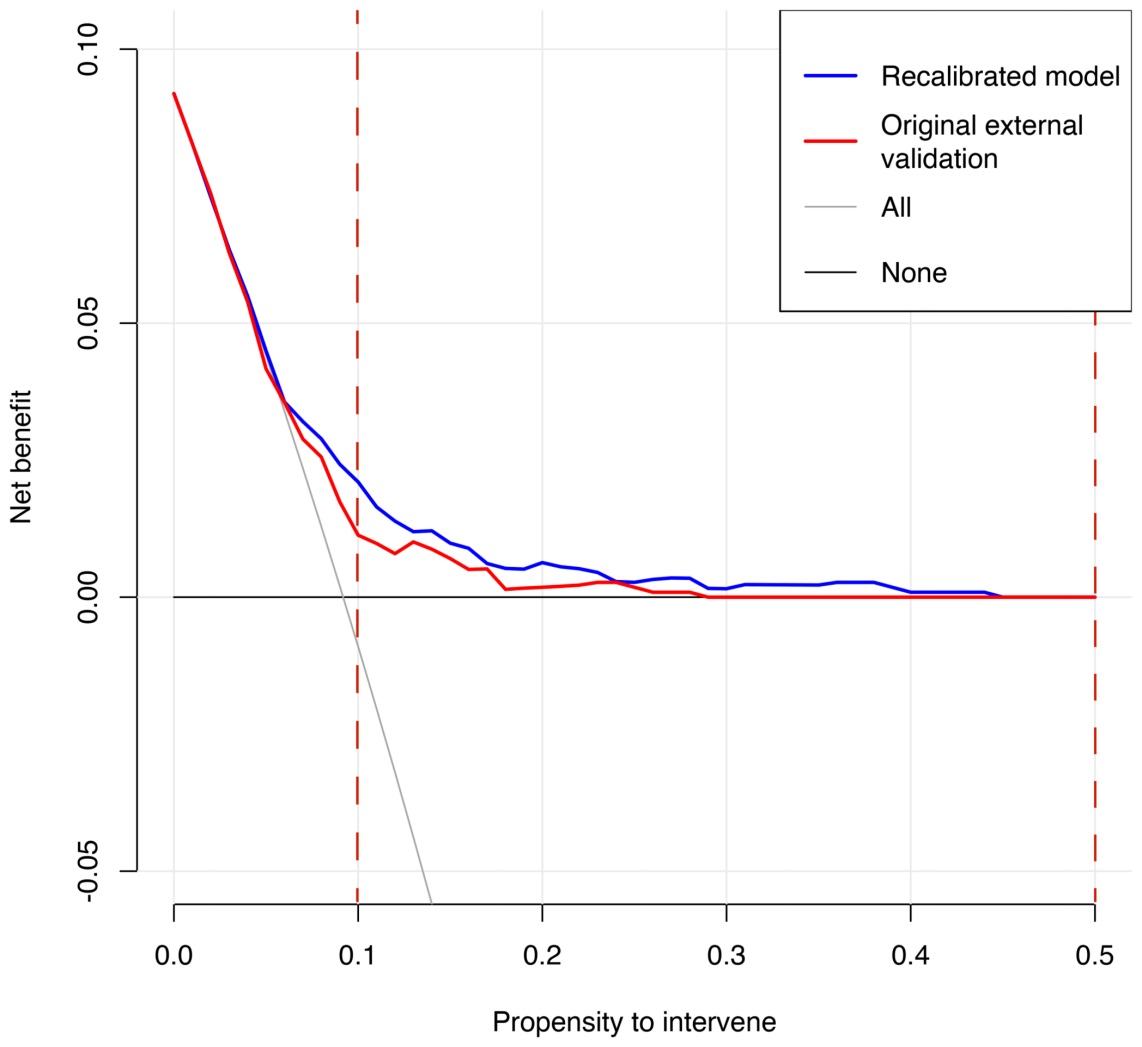
Decision curve analysis plot for forced-entry original and recalibrated models The plot reports net benefit (y axis) of forced-entry (MOZART) original and recalibrated models across a range of propensity to intervene thresholds (x axis) compared with intervening in all patients, or intervening in no patients. The shaded red vertical lines represent the two thresholds we selected *a priori* to study potential clinical value of low- and high-risk interventions (e.g., monitoring or starting clozapine).

Decision curve analysis for MOZART (Figure 3) suggests that at propensity to intervene thresholds greater than 0.05 (revised model) or 0.06 (original model), the models provided greater net benefit than the competing extremes of treating all patients or none. The recalibrated model provided higher net benefit at most, if not all, thresholds over 0.05 than the original model.

Numerical decision curve analysis results (net benefit, standardised net benefit, sensitivity, and specificity) are shown in Supplementary Table 4 across a range of propensity to intervene thresholds. For example, if a low-risk intervention such as close monitoring for TRS was considered suitable above a propensity to intervene threshold of 0.10 (>10% risk of clozapine use), the recalibrated model would provide a net benefit of 2% (95% CI 1-4%), meaning that an additional 24% of patients could be closely monitored for the presence of TRS (standardised net benefit). However, for a potentially more invasive intervention such as starting clozapine treatment, at apropensity to intervene threshold of 0.50, the same model would provide no net benefit, due to insufficient sensitivity.

We also developed an online data visualisation tool for both the original and recalibrated MOZART models, which allows to interactively explore the effect of each predictor and their combinations on the risk of clozapine use based on the predictors included in this study.See https://eosimo.shinyapps.io/trs_app/

### Sensitivity analysis: iterative improvements versions of the forced-entry model

Model 1 (M1) comprised sex as the only predictor; M2 included all demographics; M3 included all demographics, plustriglyceride levels; M4 included all the above plus ALP.The internal coefficients and shrinkage factors for each model are presented in Supplementary Table 5. The C statistic increased from 0.56 (95%CI: 0.50-0.62) for M1 to 0.69 (95%CI: 0.62-0.76) for M4. Calibration plots showed good agreement between observed and expected risk at most predicted probabilities for M3 and M4 (shown, alongside histograms of predicted outcome probabilities, in Supplementary Figures 6 to 9).

## Discussion

We examined the predictive potential of routinely collected and readily available sociodemographic, lifestyle, and clinical information, obtained at the start of a first psychosis episodefor the risk of clozapine use, as a proxy for developing treatment-resistant schizophrenia (TRS). We developed two models, one, MOZART, based on forced-entry logistic regression, and one based on LASSO for coefficient generation and shrinkage. MOZART used manually pre-selected candidate biological predictors of clozapine use, based on previous literature, clinical availability, and rationale. The two models performed adequately both in internal and external validation. MOZART performed better than LASSO at external validation, possibly because it was more parsimonious (using seven predictors instead of eleven), thus reducing the risk of model overfitting. MOZART’s performance in external validation improved following logistic recalibration and model updating.

Decision curve analysis revealed that MOZARTshows clinical utility at lower propensity to intervene thresholds, such as between 10 and 20%. This model cannot yet be recommended for clinical use and requires prospective validation in larger samples, health technology assessment, and regulatory approval. However, subject to these steps, in future our model could allow to implement low-risk strategies, e.g., stratifying patients at higher-than-average risk of developing antipsychotic resistance for closer psychiatric monitoring for the presence of TRS. These strategies have very low, if any, risk of causing harm, and might show potential at earlier recognition and treatment of TRS. Clozapine is more effective when given soon after treatment resistance is established, although in clinical practice there are long delays to starting it^7, 8^; therefore, starting treatment early might show potential inreducing symptoms and improving quality of life in people with unrecognised TRS.

However, given the higher risk and licensing conditions of clozapine, and the lower sensitivity of the model at higher risk thresholds, this model alone will not be useful for initiating higher-risk interventions, such as starting clozapine.

In sensitivity analyses we also explored the incremental value of models based on only one, four, five, or six predictors, and found incremental predictive improvements when adding commonly recorded biological markers, suggesting their potential usefulness in future psychosis prediction studies.

In future, the inclusion of genetic risk scores might make clozapine prediction models more accurate, and therefore more clinically useful. Two existing studies found that polygenic risk scores for schizophrenia did not produce significant increases in predictive power of a model for TRS^17, 39^. However, the publication since then of larger genome-wide association studies (GWAS) for schizophrenia^40^ and of a specific TRS GWAS^41^ will likely make the approach more powerful. However, at the current level of availability of genotyping or sequencing to clinical samples, this approach is not currently feasible, if not in selected research settings.

The present study is innovative in creating a prediction model for clozapine use based only on routinely measured clinical and demographic information, including biomarkers, available at FEP baseline, as per PROBAST criteria^42^. MOZARTperforms similarly to existing research in the field – which included a larger number of predictors, of which some are not commonly recorded in clinical practice^10^ – and shows clinical usefulness, despite being based on just seven routinely collectedpredictors. In addition, we extend upon existing research by including an external validation analysis, a crucial step to demonstrate likely generalizability, and followingbest practice guidelines^42, 43^, as recently done for similar outcomes^44^.

We show that simple blood-based biomarkers measured at the onset of psychosis can explain part of the variance of the risk of clozapine use: MOZART’s C statistic (including triglycerides, ALP and lymphocyte counts) was greater than that of the demographics-only model in internal validation. This suggests that the variance of a psychiatric phenotype (resistance to antipsychotic medication) may be explained, at least in part, by inflammatory, fat, and liver biomarkers measured at FEP onset.

Previous studies using regression-based methods have shown that elevated triglycerides are associated with a worse psychiatric clinical outcome in psychosis at the group level^2, 27^. We extend these findings by showing that elevated triglycerides at the individual level could aid in prediction of clozapine use. We included ALP due to the increasing importance that liver dysfunction is thought to play in the psychosis spectrum^29^. In particular, elevated ALP might relate to the primary dysglycaemic and dysmetabolic phenotype of FEP^24, 45, 46^, or it might be its consequence (hyperlipidaemia leading to NAFLD^28^, a phenotype which has been found in FEP^29^). Elevated ALP may also capture some of the variance of substance use in a more objective manner than self-report^47, 48^.

Regarding inflammatory markers, we chose to use lymphocyte count because of data availability. In a previous analysis (of a group of mostly White European participants), lymphocytes were elevated in the FEP sub-group with a worse psychiatric outcome^2^; however, cross-sectional studies have not found lymphocyte elevations in FEP^49, 50^, and a recent Mendelian randomisation study did not find evidence for a causal association with schizophrenia^51^, potentially discounting the likelihood of a causal association of elevated lymphocytes with schizophrenia in general. Further, we found that the drop in discrimination performance for the forced-entry model from internal to external validation was mostly due to differences in the lymphocyte predictor, with the sign of the coefficient switching direction between samples. In model updating,the C-statistic could be partially preserved by updating the coefficient for lymphocytes. This might be explained by the different ethnic mix between the development sample (mainly White ethnicity) and the external validation sample (mainly Black African/Caribbean ethnicity). It is well known that inflammatory markers, including lymphocytes, show different distributions in different ethnic groups^52, 53^. This might encourage repeating the analysis using different inflammatory markers, such as CRP, in future research. We could not include CRP since in the included cohorts it was most often sampled when there was suspicion of infection; therefore, data was available only for a small subset, and likely showing strong selection bias.

Table 2 shows that performance in external validation increased following logistic recalibration of the model; differences in prevalence of clozapine use between the development and external validation samples may partly explain this. Given our pragmatic definition of TRS, based on clozapine treatment, this prevalence difference might be due to differences in clinician attitudes to medication, case mix (including severity and ethnicity), or other local differences.

### Strengths and limitations

The use of longitudinal EIS cohort data is the main strength of this study. Enrolment into an EIS fosters confidence in the psychiatric phenotype of included participants, and into the naturalistic nature of the sample. Specifically, the CAMEO EIS, used for development of our model, accepts people presenting with confirmed psychotic symptoms from any cause, including drug induced psychoses and affective psychoses (including ICD-10 codesF06.0-2, F20-F31, F32.3, F33.3, F53.1); therefore, MOZARTis shown to work in a real-life sample of FEP, which will predisposes the results to be more clinically applicable (i.e., to any patient presenting with a FEP). Another strength of this study is the naturalistic study design, including a large number of consecutive referrals with little possibility of selection bias from the sampling frame. Most EISs in the UK NHS, including all three in this analysis, are the only treatment providers for FEP in a given geographical area, thus covering a large proportion of all incident cases of first-episode psychosis in a defined catchment area. Because this study is based on real-life patient data from EHRs from different regions, we were unable to address potential secular and regional trends in monitoring, laboratory testing and prescribing practice that could have biased results. However, in doing so we adhered to best prediction modelling practice, which requires external validation on separate participants, or risk “high risk of bias”^42^. Furthermore, we used routinely measured, clinically available blood-based biomarkers, which warrant a high confidence in the validity of the measures, as well as aiding the potential clinical translation of our findings.

Among the limitations of this study, we used clozapine treatment as the outcome, i.e. as a proxy measure for TRS, as in several previous studies^14^. Prevalence of clozapine use inour samples was lower than the expected prevalence of 13% (see calculation in the Methods/Outcome section).In the UK, clozapine should be offered to all patients with TRS^30^. However, a recent national audit showed that only 52% of patients with FEP who have not responded adequately to at least 2 antipsychotics are offered clozapine^54^. Furthermore,as mentioned, EIS services accept patients with psychotic symptoms from any cause, thus including, for example, bipolar and unipolar mood disorders; this diagnostically inclusive nature of our FEP cohort might partially explain the relatively low rate of TRS. However, while our outcome definition may have a reduced sensitivity for capturing treatment resistance, the specificity is likely to be high; indeed, the UK National Institute for Health and Care Excellence (NICE) guidance is that prescription of clozapine is reserved for those with schizophrenia in whom two trials of antipsychotics have failed (including one second-generation antipsychotic)^55^,and the only UK indication for clozapine other than TRS is Parkinson’s disease, which would be extremely rare in a FEP cohort only including adults up to 65 (mean age of 28/29 years, as per Table 1). Further, the literature suggests that clozapine in the UKis used *off label* for treating refractory mania, psychotic depression, aggression in psychotic patients, the reduction of tardive dyskinesia symptoms and borderline personality disorder^56^, therefore the presence of a few such diagnoses among the cases cannot be excluded, and is a limitation of this study. However, a UK-based systematic investigation of *off label* antipsychotic use in secondary care established that clozapine is the least likely to be used outside its approved indications, with only one of 502 patients (~2%) in the study using it *off label*^57^, which might be a consequence of the very strict regulations in place for clozapine use. Another UK-based study of TRS, including 14,299 patients, both inpatient and community-based, undergoing mandatory clozapine blood-monitoring, found 56 *off label* clozapine prescriptions, or 0.4%^58^. While these studies included any patient on antipsychotics, our cohorts are based on UK EIS teams, which are commissioned to only accept young patients with a first episode of psychosis (and not with personality disorders), and therefore it is likely that *off label* clozapine use in this group is even rarer.

Further, not all cohorts could provide information about time of clozapine initiation, and therefore time-to-event analysis could not be performed. Moreover, follow-up data was available for up to 8 years following a FEP; this means that we might not have been able to capture “late onset” TRS, which might develop after a number of relapses, and over a number of years^59^; this might also help to explain the relatively low clozapine rate in our samples. Predictor availability was limited to those markers that were available in all three study cohorts. No cohort included a symptom or severity measure, such as the Positive and Negative Syndrome Scale (PANSS); we could therefore not include symptoms at baseline as a predictor. However, systematic assessment and recording of symptoms using standardised assessment tools is unfortunately uncommon in UK EIS, and therefore this would not have been listed under the included “routinely collected and readily available” predictors. The number of predictors that we could include was also limited by our sample size, although we took particular care in predictor selection and this may have helped to prevent model overfitting^32, 43^. It must be pointed out that this work did not aim to make any assumptions about whether the included predictors might be causal to TRS: variables were selected if they were known to be associated – i.e., likely capturing part of the outcome’s variance.

Further, we used bootstrap resampling to obtain an estimate of the corrected calibration slope, which was then used as a penalty factor for our coefficients to reduce the risk of over-fitting; bootstrapping can be limited in samples of rare events, however its use is preferable to using the original coefficients to reduce the risk of overfitting^42^.

A further limitation of this work is the potential for the inclusion of patients already taking antipsychotic medication at baseline. Antipsychotics could influence the levels of the biomarkers. However, most patients admitted to an EIS are medication naïve or minimally treated. Bloods tests were only used for prediction if performed within 100 days of referral to the EIS; it is likely that some patients were started on antipsychotic medication during this time, though the duration of treatment is likely to have been relatively short. However, participants were excluded if the outcome (starting clozapine) pre-dated baseline blood collection.

In conclusion, we report that, based on three large samples of FEP patients, routinely recorded demographics and biomarkers measured at presentation with a FEP could be useful in the individualized prediction of the risk of clozapine use (as a proxy for developing TRS) up to eight years later. Subject to further external validation and regulatory approval, MOZART appears useful at predicting the risk of TRS at lower propensity to intervene thresholds, thus potentially allowing to implement low-risk strategies such as closer psychiatric monitoring for TRS in at-risk populations.This could potentially speed up the time from FEP onset to clozapine start, thus reducing delays in TRS recognition and treatment, and consequently reducingsuffering and improving quality of life.

We suggest that future efforts in TRS risk prediction should seek to consider such routinely collected data. Doing so may improve both model predictive performance and likely clinical usefulness, both of which are crucial for the future routine deployment of a risk prediction model into clinical practice.

## Supplementary Material

Supplementary methods, tables and figures

## Figures and Tables

**Table 1:** Predictor Comparisons between Samples Used in Model Development and Internal/External Validation

Predictor	Sample			
	Development			External Validation
	CAMEO EIS	Birmingham EIS	Pooled Development Sample	SLaM EIS Validation Sample
Final Included Sample size, *N*.	539	246	785	1,110
Male Sex, *N*. (%)	328 (60.9%)	146 (59.3%)	474 (60.4%)	692 (62.3%)
Age in Years, mean (SD) min, max	30.23 (12.00) 14, 65	23.86 (4.87) 15, 37	28.24 (10.73) 14, 65	28.82 (9.94) 17.5, 64
White/unrecorded Ethnicity, *N*. (%)	449 (83.3%)	70 (28.4%)	519 (66.1%)	378 (34.0%)
Black/African-Caribbean Ethnicity, *N*. (%)	21 (3.9%)	57 (23.2%)	78 (9.9%)	507 (45.7%)
Asian Ethnicity, *N*. (%)	69 (12.8%)	119 (48.4%)	188 (23.9%)	225 (20.3%)
Triglycerides, mmol/L, mean (SD)	1.42 (1.07)	1.55 (1.30)	1.46 (1.15)	1.25 (0.96)
Alkaline phosphatase (ALP), U/L, mean (SD)	78.58 (25.55)	82.67 (25.78)	79.86 (25.68)	75.03 (22.01)
Lymphocyte count, billion/L, mean (SD)	1.91 (0.69)	2.22 (0.79)	2.01 (0.74)	1.98 (0.64)
Smoking, *N*. (%)	201 (37.3%)	124 (50.4%)	325 (41.4%)	468 (42.2%)
Body mass index (BMI), kg/m2, mean (SD)	24.68 (6.65)	25.74 (5.78)	25.01 (6.41)	24.07 (5.58)
Random plasma glucose (mmol/L), mean (SD)	5.25 (1.73)	4.87 (1.28)	5.13 (1.61)	5.11 (1.80)
Neutrophil count, billion/L, mean (SD)	4.60 (2.00)	4.14 (2.06)	4.46 (2.03)	4.01 (1.98)
Follow-up time, years, mean (SD) min, max	4.45 (1.57) 0.58*, 8.50	3.55 (0.58) 2.67, 4.58	4.17 (1.40)	4.41 (1.76) 0.75*, 8.75
TRS at Follow-up, *N*. (%)	35 (6.5%)	23 (9.3%)	58 (7.4%)	102 (9.2%)

SLaM, South London and Maudsley NHS Foundation Trust; EIS, Early Intervention Service; CAMEO, Cambridgeshire and Peterborough Assessing, Managing and Enhancing Outcomes EIS (Cambridgeshire and Peterborough Foundation NHS Trust); SD, standard deviation; TRS, treatment-resistant schizophrenia.

* only TRS cases have been included if they had follow-up < 2 years

**Table 2:** Model comparisons including coefficients for development and external validation

Model type	Model Predictors of TRS	Coefficients after shrinkage for optimism$	Pooled Development Sample performance statistics	Shrinkage factor	Validation Sample performance statistics	Validation Sample New coefficients after model recalibration	Validation Sample Performance after model recalibration	Calibration plots for external validation
Forced-entry	*Intercept* Sex Age Black/African-Caribbean ethnicity Asian ethnicity Triglycerides Alkaline phosphatase (ALP) Lymphocyte count	*-2.827381* 0.286466741 -0.036205346 0.419614174 -0.144147329 0.149214138 0.006713513 0.131215526	C: 0.70 (0.63-0.76) Brier score: 0.07	0.79	C: 0.63 (0.58-0.69) Brier score: 0.08	Lymphocyte coefficient: -0.695404405. *Intercept: -1.336220* *Slope: 1.0519963*	C: 0.67 (0.62-0.73) Brier score: 0.08	Figure 2A and 2B
LASSO-based	*Intercept* Sex Age Black/African-Caribbean ethnicity Asian ethnicity Triglycerides Alkaline phosphatase (ALP) Lymphocyte count Smoking status Body mass index (BMI) Random plasma glucose Neutrophil count	*-2.736365* 0.132050 -0.248397 0.304147 -0.002375 0.139795 0.131153 0.060623 0.057593 -0.026467 -0.027369 -0.012826	C: 0.69 (0.63-0.77) Brier score: 0.07	N/A	C: 0.64 (0.58-0.69) Brier score: 0.08	Lymphocyte coefficient: -0.03608036. *Intercept: -2.706553* *Slope: 1.3102021*	C: 0.64 (0.58-0.69) Brier score: 0.08	Figure 2C and 2D

C, C value (95% confidence interval) (see Methods).

$ The coefficients are relative to non-scaled values for forced-entry models, and to scaled and centered values for the LASSO model.

## Data Availability

The source data for this work is anonymised patient records, securely held on clinical systems and available to qualified applicants following ethical approval. Therefore, the raw data cannot be shared widely. However, we developed an online data visualisation tool for both the original and recalibrated MOZART models, which allows to interactively explore the effect of each predictor and their combinations on the risk of clozapine use based on the predictors included in this study. See https://eosimo.shinyapps.io/trs_app/

## Code availability

R code for data extraction and analysis is available upon request to the Corresponding author.
