# Supplementary material for: Predicting treatment resistance from first-episode psychosis using routinely collected clinical information
Source: Nat Ment Health. Author manuscript; Available in PMC 2023 Apr 7. (PMC7614410; doi:10.1038/s44220-022-00001-z)
Supplement: Supplementary methods, tables and figures [file EMS160228-supplement-Supplementary_methods__tables_and_figures.pdf]

Development and external validation of a risk prediction model for treatment-resistant schizophrenia in people with first-episode psychosis using routinely collected clinical information

Osimo & Perry et al, 2022

Supplementary Materials

Table of contents:

Supplementary Methods **2**
Development Sample 2
Sample Size Calculation for Development of the Prediction Algorithm 2
Missing Data 3
Rationale for selection and coding of predictors 4
Model recalibration 7

Supplementary tables **8**
Supplementary Table 1: TRIPOD Checklist: Prediction Model Development and Validation 8
Supplementary Table 2: Comparison of total and included samples 9
Supplementary Table 3: Univariate associations 10
Supplementary Table 4: Decision Curve Analysis Results at Different Risk Propensity Thresholds – Forced-entry Model 11
Supplementary Table 5: Model comparisons including coefficients for development and internal validation 13

Supplementary Figures **15**

Supplementary References **21**

Supplementary Methods

Development Sample

CAMEO data were identified by anonymously searching for all EIS patients enrolled between 2013-01-01 and 2021-05-31 using the CPFT Research Database[1] (UK National Health Service [NHS] Research Ethics Service references 12/EE/0407, 17/EE/0442).

Anonymised data for all patients enrolled in the Birmingham EIS were collected between 2014-01-01 and 2018-12-31 as part of the National Clinical Audit of Psychosis Quality Improvement Programme, and were enhanced locally with biomarker data; the work conformed to the Health Research Authority definition of service evaluation (confirmed by Birmingham Women's and Children's Hospital NHS Foundation Trust).

Patients were excluded if they died or moved out of the Trust's catchment area during follow-up.

Sample Size Calculation for Development of the Prediction Algorithm

Riley and colleagues[2] proposed a set of criteria that sample size should meet for development of a binary outcome prediction algorithm, to minimise the risk of overfitting and to ensure precise estimation of key parameters in the prediction algorithm. The sample size calculation requires the user-specified anticipated R^2 of the algorithm, and the average outcome value and standard deviation of outcome values in the population of interest. The final recommended sample size is taken as the largest of the three individual calculations. See [2] for more information.

These criteria have been developed into a statistical package, *pmsampsize*[3] for R[4], which we used for sample size calculation. The user-specified arguments were:

1) Outcome prevalence = 0.13. The calculation: population prevalence of TRS is 23%[5-7]. We expected to capture mostly "early onset" cases, which represent ~84% of cases[8]. From previous literature, clozapine is given in ~68% of TRS cases[8], so the expected prevalence was = 0.23 * 0.84 * 0.68 = 0.13.
2) R^2 = 0.10, selected as an estimate from the most similar previous study on the topic[8].
3) Shrinkage = 0.80.

Results of Sample Size Calculations using pmsampsize[3] based on criteria proposed by Riley et al.[2]

Criteria	Sample Size	Shrinkage	Parameters	R^2	Events Per Predictor
**Full-Model**					
Criteria 1*	412	0.80	11	0.10	4.87
Criteria 2*	381	0.787	11	0.10	4.50
Criteria 3*	174	0.80	11	0.10	2.06
Final	412	0.80	11	0.10	4.87

* the three criteria were:
a) small overfitting defined by an expected shrinkage of predictor effects by 10% or less;
b) small absolute difference of 0.05 in the algorithm's apparent and adjusted Nagelkerke's R-squared value;
c) precise estimation (within +/- 0.05) of the average outcome risk in the population.

The details of the variable selection process are in the *Predictor variables* section of the main Methods.

For the forced-entry model we selected *a priori* the most relevant seven variables based on a balance of clinical knowledge, past research, and likely clinical usefulness. This is below the maximum allowed of 11 variables for our sample size, however we preferred a more parsimonious model for forced-entry because we wanted to reduce the likelihood there would be overfitting. For the LASSO model we included the maximum allowed of 11 variables, adding a few additional 'potentially relevant' variables, since LASSO includes a variable reduction step as an extra defence against overfitting.

Missing Data

We used multiple imputation using chained equations (MICE)[9] for missing data in all samples for predictors which were <50% missing[10] and had suitable auxiliary variables available for use as 'indicators of missingness' to reduce the impact of 'missing not at random' bias[11]. We imputed 100 datasets. Auxiliary variables were selected based upon minimizing the fraction of missing information[12]. Box-and-whisker and density plots were used to check similarities of observed and imputed data. Estimates were pooled using Rubin's rules.

Proportion of Missing Data per Variable for Model Development and External Validation

Variable	Model Development Sample		External Validation Sample	
	Non-cases	Cases	Non-cases	Cases
Sex	0%	0%	0%	0%
Age	0%	0%	0%	0%
Ethnicity	0%	0%	0%	0%
Triglycerides	35.9%	35.6%	12.4%	14.7%
Alkaline phosphatase	24.2%	33.3%	<1%	No missing
Lymphocyte counts	27.2%	37.8%	<1%	No missing
Neutrophil count	26.5%	37.8%	<1%	No missing
Smoking status	49.0%	64.4%	27.2%	19.6%
Body mass index (BMI)	30.0%	31.1%	21.5%	19.6%
Random glucose levels	43.6%	42.2%	44.9%	38.2%
Test	χ^2 (df=6) = 0.03, p = 1.00		χ^2 (df=6) = 0.03, p = 1.00	

The χ^2 test was performed only between variables with missing data, namely triglycerides, alkaline phosphatase, lymphocyte and neutrophil counts, smoking, BMI and glucose levels.

Rationale for selection and coding of predictors

For the CAMEO dataset, custom-built natural language processing (NLP) software[1] was used to extract numerical cardiometabolic and inflammatory marker data from unstructured text, e.g. medical notes, as previously described[13]. Some of the NLP tools for the extraction of cardiometabolic marker data were developed specifically for this study. Accuracy and reliability for all cardiometabolic markers were satisfactory, as measured by recall (probability of retrieving a record given it was relevant; >0.75 for all) and precision (probability of a record being relevant, given it was retrieved; >0.90 for all) statistics (see [14] for how these were calculated).

Variables included in the forced entry model

We created a forced entry model by selecting only commonly used clinical predictors, on a balance of clinical knowledge, past research, and likely clinical usefulness. The forced entry model was designed to include only demographics (age, sex, and ethinicity) plus one lipid homeostasis, one inflammatory, and one liver function marker.

Sex

Sex is frequently considered in TRS risk prediction algorithms[15], and so we included it in ours. One previous paper considered interactions between male sex and other predictors (age)[5]. We did not take this step in order to limit potential model complexity and thus reduce the risk of model overfitting given our sample size, and reduce the risk of hampering external validation performance and thus generalizability[16]. There are well-known sex differences in the epidemiology, aetiology, biology and clinical expression of psychosis[17]. Recent

meta-analytic reports have suggested that male sex is an important risk factor for developing TRS[15]. Considering our available sample size, we did not consider separate algorithms for males and females, and so chose to model sex as a binary variable.

Age

Age is the most frequently included predictor in existing TRS risk prediction algorithms[15], and we also included it in our algorithm as a continuous variable. One previous paper considered interactions between age and other predictors (sex and ethnicity)[5]; to our knowledge, no previous work used age as a non-linear term. We did not take this step in order to limit potential model complexity and thus reduce the risk of model overfitting given our sample size, and reduce the risk of hampering external validation performance and thus generalizability[16].

Ethnicity

Ethnicity is included in existing TRS risk prediction algorithms[15]. Black ethnicity was found to be a significant predictor of TRS in[5], while it was not significant in[8]. One previous paper considered interactions between Black ethnicity and other predictors (age) [5]. We did not take this step in order to limit potential model complexity and thus reduce the risk of model overfitting given our sample size, and reduce the risk of hampering external validation performance and thus generalizability[16]. In our development and validation samples, ethnicity was recorded inconsistently, with the majority of included records classified in relatively simple terms, for example "White", or "Asian". However, these simplified classifications do not recognise the heterogeneity which may exist within these groupings, therefore potentially incorrectly implying that the populations are homogeneous. Nevertheless, to strike an appropriate balance between our available sample size, the case mix of our development and validation samples, and with a consideration to maximise coding harmonisation between datasets, we proceeded with a categorical nominal variable with as much granularity as the data permitted, and so our variable consisting of White European/not stated (reference category), Black/African-Caribbean ethnicity, and Asian/Other ethnicity.

Triglyceride levels

Triglyceride levels are frequently included in cardiometabolic risk prediction algorithms for people with psychosis[18]. They also prominently feature in the Psychosis Metabolic Risk Calculator (PsyMetRiC) that we have recently developed[19].

Previous research has shown that triglycerides, if elevated, may be longitudinally associated with adverse clinical outcomes, i.e. a more chronic illness course[13, 20].

They are also commonly recorded in clinical practice and correlate well with other measures of the metabolic syndrome[21]. Therefore, we included triglycerides as a continuous variable.

Alkaline phosphatase (ALP) levels

People with schizophrenia have been shown to present with increased prevalence of multiple metabolic conditions, including non-alcoholic fatty liver disease (NAFLD)[22, 23], a condition that occurs when lipids accumulate in the liver[24]. NAFLD can be a consequence of other immune and metabolic dyscrasias that associate with schizophrenia, namely a pro-inflammatory state, hypertriglyceridemia and altered diets.

To our knowledge, this is the first time that ALP levels have been studied in schizophrenia as a predictor of psychiatric outcome.

We have included this marker in our forced entry model (as the "liver function" measure).

Lymphocyte and neutrophil cell counts

The best studied inflammatory marker in psychosis is C-reactive protein. However, this is not routinely measured at FEP onset if the patient does not present with inflammatory features, such as a temperature. Therefore, we decided against using this measure and we selected inflammatory cell count measures instead. Lymphocyte and neutrophil cell counts are commonly measured at disease onset as part of a full blood count, and have been associated with psychosis in cross sectional studies[25, 26]. Furthermore, when elevated they have been found to associate with worse disease psychiatric outcomes[13]. Therefore, we have included the best studied, lymphocyte count, in our forced entry model (as the "immune" measure), and the other (neutrophil count) in our LASSO model.

Variables included in the LASSO model

We included a further four variables in our LASSO model, which have less evidence for an effect on outcomes in psychosis but are all commonly recorded clinical variables at FEP onset. One, neutrophil count, is described above.

Smoking status

Daily tobacco use has been associated with the risk of psychosis in a recent large meta-analysis[27]. Despite this, tobacco use has not been included in any of the existing TRS prediction algorithms[15]. Given the inconsistent recording of smoking at FEP onset across different databases, and to strike an appropriate balance with our available sample size, we proceeded with a categorical nominal variable consisting of non-smoker (reference category) versus smoker (at least 1 cigarette per day).

Body Mass Index

Body mass index (BMI) has been shown to be an important risk factor to predict the development of the metabolic syndrome in FEP in the Psychosis Metabolic Risk Calculator (PsyMetRiC) that we have recently developed[19]. In our analysis, BMI has not been reliably shown to associate with psychiatric outcomes[13], even if it was associated with a worse 1-year outcome in one study[20], so we did not include it in our forced-entry model. It is commonly recorded in clinical practice. Therefore, we included it as a continuous variable.

Random glucose levels

People with FEP show insulin resistance, defined as high insulin for a given glucose level[28]. Recently, changes in insulin sensitivity and adiposity starting from childhood were shown to have potential disorder-specific associations with psychosis[29]. Unfortunately, insulin levels are not routinely measured in FEP. Therefore, we decided to use random glucose levels as an imperfect proxy of glucose control.

In a recent paper we did not find any association between glucose levels at FEP onset and long-term psychiatric outcomes[13]. Given this, and the imperfect nature of glucose as a proxy for insulin sensitivity, this marker was not included in our forced entry model.

Model recalibration

To perform logistic recalibration, we fitted a model using the linear predictor of the original model as a single predictor in the external validation sample. The model was then updated by multiplying the linear predictor by the coefficient and adding the estimated intercept; the individual risks were then recalculated, and predictive performance re-assessed using the methods described above.

Supplementary tables

Supplementary Table 1: TRIPOD Checklist: Prediction Model Development and Validation

Section/Topic			Checklist Item	Page
**Title and abstract**				
Title	1	;V	Identify the study as developing and/or validating a multivariable prediction model, the target population, and the outcome to be predicted.	2
Abstract	2	;V	Provide a summary of objectives, study design, setting, participants, sample size, predictors, outcome, statistical analysis, results, and conclusions.	2
**Introduction**				
Background and objectives	3a	;V	Explain the medical context (including whether diagnostic or prognostic) and rationale for developing or validating the multivariable prediction model, including references to existing models.	3-4
	3b	;V	Specify the objectives, including whether the study describes the development or validation of the model or both.	4
**Methods**				
Source of data	4a	;V	Describe the study design or source of data (e.g., randomized trial, cohort, or registry data), separately for the development and validation data sets, if applicable.	5-6
	4b	;V	Specify the key study dates, including start of accrual; end of accrual; and, if applicable, end of follow-up.	5-6
Participants	5a	;V	Specify key elements of the study setting (e.g., primary care, secondary care, general population) including number and location of centres.	5-6
	5b	;V	Describe eligibility criteria for participants.	5-6
	5c	;V	Give details of treatments received, if relevant.	N/A
Outcome	6a	;V	Clearly define the outcome that is predicted by the prediction model, including how and when assessed.	6
	6b	;V	Report any actions to blind assessment of the outcome to be predicted.	N/A
Predictors	7a	;V	Clearly define all predictors used in developing or validating the multivariable prediction model, including how and when they were measured.	6-7 + suppl
	7b	;V	Report any actions to blind assessment of predictors for the outcome and other predictors.	N/A
Sample size	8	;V	Explain how the study size was arrived at.	7 + suppl
Missing data	9	;V	Describe how missing data were handled (e.g., complete-case analysis, single imputation, multiple imputation) with details of any imputation method.	7 + suppl
Statistical analysis methods	0a	D	Describe how predictors were handled in the analyses.	7 + suppl
	0b	D	Specify type of model, all model-building procedures (including any predictor selection), and method for internal validation.	7-8
	0c	V	For validation, describe how the predictions were calculated.	7-8
	0d	;V	Specify all measures used to assess model performance and, if relevant, to compare multiple models.	8
	0e	V	Describe any model updating (e.g., recalibration) arising from the validation, if done.	8-9
Risk groups	11	;V	Provide details on how risk groups were created, if done.	N/A
Development vs. validation	12	V	For validation, identify any differences from the development data in setting, eligibility criteria, outcome, and predictors.	6
**Results**				
Participants	3a	;V	Describe the flow of participants through the study, including the number of participants with and without the outcome and, if applicable, a summary of the follow-up time. A diagram may be helpful.	10 (and 5)
	3b	;V	Describe the characteristics of the participants (basic demographics, clinical features, available predictors), including the number of participants with missing data for predictors and outcome.	T1
	3c	V	For validation, show a comparison with the development data of the distribution of important variables (demographics, predictors and outcome).	T1
Model development	4a	D	Specify the number of participants and outcome events in each analysis.	T1
	4b	D	If done, report the unadjusted association between each candidate predictor and outcome.	N/A
Model specification	5a	D	Present the full prediction model to allow predictions for individuals (i.e., all regression coefficients, and model intercept or baseline survival at a given time point).	T2
	5b	D	Explain how to the use the prediction model.	T2
Model performance	16	;V	Report performance measures (with CIs) for the prediction model.	T2
Model-updating	17	V	If done, report the results from any model updating (i.e., model specification, model performance).	T2
**Discussion**				
Limitations	18	;V	Discuss any limitations of the study (such as nonrepresentative sample, few events per predictor, missing data).	18-19
Interpretation	9a	V	For validation, discuss the results with reference to performance in the development data, and any other validation data.	16
	9b	;V	Give an overall interpretation of the results, considering objectives, limitations, results from similar studies, and other relevant evidence.	16-17
Implications	20	;V	Discuss the potential clinical use of the model and implications for future research.	20
**Other information**				
Supplementary information	21	;V	Provide information about the availability of supplementary resources, such as study protocol, Web calculator, and data sets.	in each section
Funding	22	;V	Give the source of funding and the role of the funders for the present study.	21-22

Supplementary Table 2: Comparison of total and included samples

	Development				External Validation	
	**Total CAMEO EIS**	**Included CAMEO EIS**	**Total Birmingham EIS***	**Included Birmingham EIS**	**Total SLaM EIS**	**Included SLaM EIS**
Sample size, *N*.	1,660	539	1,913	246	3,144	1,110
Male Sex, *N*. (%)	1,008 (60.7%)	328 (60.9%)	1,259 (65.8%)	146 (59.3%)	1,913 (60.8%)	692 (62.3%)
Age in Years, mean (SD)<br>min, max	26.08 (9.8)<br>13, 71	30.23 (12.00)<br>14, 65	23.04 (4.95)<br>13, 37	23.86 (4.87)<br>15, 37	29.05 (10.04)<br>17, 64.5	28.82 (9.94)<br>17.5, 64
White/unrecorded Ethnicity, *N*. (%)	1,427 (86.0%)	449 (83.3%)	N/A	70 (28.4%)	1,199 (38.1%)	378 (34.0%)
Black/African-Caribbean Ethnicity, *N*. (%)	58 (3.5%)	21 (3.9%)	N/A	57 (23.2%)	1,337 (42.5%)	507 (45.7%)
Asian Ethnicity, *N*. (%)	175 (10.5%)	69 (12.8%)	N/A	119 (48.4%)	608 (19.3%)	225 (20.3%)

* Total numbers are an estimate based on Ns between 2016 and 2018
N/A: not available

Supplementary Table 3: Univariate associations

Logistic regressions based on original, non-imputed datasets

	CAMEO EIS	Birmingham EIS	SLaM EIS
Male sex	Wald Z (N=539)=2.62, p=0.009<br>R^2=0.041, Brier=0.060	Wald Z (N=246)=-1.24, p=0.214<br>R^2=0.022, Brier=0.039	Wald Z (N=1,110)=2.42, p=0.016<br>R^2=0.012, Brier=0.083
Age	Wald Z (N=539)=-2.48, p=0.013<br>R^2=0.037, Brier=0.060	Wald Z (N=246)=-0.17, p=0.862<br>R^2=0.000, Brier=0.039	Wald Z (N=1,110)=-4.38, p<0.001<br>R^2=0.05, Brier=0.082
Black/African-Caribbean ethnicity	Wald Z (N=539) =1.43, p=0.152<br>R^2=0.008, Brier=0.060	Wald Z (N=246)=-0.97, p=0.334<br>R^2=0.017, Brier=0.039	Wald Z (N=1,110)=0.29, p=0.77<br>R^2=0.00, Brier=0.083
Asian ethnicity	Wald Z (N=539) =-0.25, p=0.802<br>R^2=0.000, Brier=0.061	Wald Z (N=246)=0.74, p=0.457<br>R^2=0.008, Brier=0.039	Wald Z (N=1,110)=0.34, p=0.73<br>R^2=0.00, Brier=0.083
Triglycerides	Wald Z (N=272) =0.90, p=0.371<br>R^2=0.007, Brier=0.065	Wald Z (N=231)=2.07, p=0.039<br>R^2=0.049, Brier=0.041	Wald Z (N=1,110)=1.48, p=0.14<br>R^2=0.004, Brier=0.081
Alkaline phosphatase (ALP)	Wald Z (N=351) =1.91, p=0.056<br>R^2=0.026, Brier=0.058	Wald Z (N=240) =-0.07, p=0.948<br>R^2=0.000, Brier=0.032	Wald Z (N=1,110)=3.02, p=0.002<br>R^2=0.002, Brier=0.083
Lymphocyte count	Wald Z (N=325) =0.35, p=0.728<br>R^2=0.001, Brier=0.052	Wald Z (N=242) =1.44, p=0.151<br>R^2=0.025, Brier=0.039	Wald Z (N=1,110)=-2.93, p=0.0034<br>R^2=0.018, Brier=0.083

Supplementary Table 4: Decision Curve Analysis Results at Different Risk Propensity Thresholds – Forced-entry Model

In bold, examples from the main text

Propensity threshold	Sensitivity Recalibrated model	Specificity Recalibrated model	Net benefit Recalibrated model	Standardised net benefit Recalibrated model	Sensitivity Original model	Specificity Original model	Net benefit Original model	Standardised net benefit Original model
0	1 (1,1)	0 (0,0)	0.09 (0.08,0.11)	1 (1,1)	1 (1,1)	0 (0,0)	0.09 (0.08,0.11)	1 (1,1)
0.01	1 (1,1)	0 (0,0.01)	0.08 (0.07,0.1)	0.9 (0.88,0.92)	1 (1,1)	0 (0,0)	0.08 (0.07,0.1)	0.9 (0.88,0.92)
0.02	0.99 (0.96,1)	0.03 (0.02,0.04)	0.07 (0.06,0.09)	0.79 (0.74,0.83)	1 (1,1)	0.02 (0.01,0.02)	0.07 (0.06,0.09)	0.8 (0.76,0.83)
0.03	0.97 (0.93,1)	0.07 (0.06,0.09)	0.06 (0.05,0.08)	0.69 (0.61,0.75)	0.97 (0.93,0.99)	0.06 (0.05,0.08)	0.06 (0.05,0.08)	0.68 (0.6,0.73)
0.04	0.95 (0.9,0.99)	0.14 (0.12,0.16)	0.05 (0.04,0.07)	0.6 (0.5,0.67)	0.94 (0.89,0.98)	0.14 (0.12,0.17)	0.05 (0.04,0.07)	0.59 (0.47,0.65)
0.05	0.89 (0.82,0.94)	0.22 (0.19,0.24)	0.04 (0.03,0.06)	0.49 (0.37,0.56)	0.85 (0.78,0.93)	0.24 (0.21,0.27)	0.04 (0.03,0.06)	0.46 (0.34,0.54)
0.06	0.83 (0.75,0.9)	0.33 (0.3,0.36)	0.04 (0.02,0.05)	0.41 (0.27,0.5)	0.79 (0.71,0.87)	0.37 (0.34,0.4)	0.04 (0.02,0.05)	0.39 (0.27,0.49)
0.07	0.79 (0.7,0.87)	0.42 (0.38,0.45)	0.03 (0.01,0.05)	0.36 (0.18,0.46)	0.69 (0.58,0.77)	0.49 (0.46,0.52)	0.03 (0.01,0.04)	0.31 (0.12,0.41)
0.08	0.73 (0.63,0.81)	0.51 (0.48,0.54)	0.03 (0.01,0.04)	0.31 (0.12,0.42)	0.61 (0.51,0.7)	0.59 (0.56,0.62)	0.02 (0.01,0.04)	0.26 (0.11,0.37)
0.09	0.65 (0.56,0.74)	0.6 (0.57,0.63)	0.02 (0.01,0.04)	0.26 (0.1,0.37)	0.5 (0.39,0.59)	0.69 (0.67,0.73)	0.02 (0,0.03)	0.2 (0.04,0.32)
**0.10**	**0.62 (0.52,0.72)**	**0.66 (0.62,0.68)**	**0.02 (0.01,0.04)**	**0.24 (0.08,0.36)**	0.37 (0.28,0.47)	0.77 (0.74,0.8)	0.01 (0,0.02)	0.12 (-0.01,0.22)
0.11	0.54 (0.45,0.64)	0.71 (0.68,0.74)	0.02 (0,0.03)	0.19 (0.06,0.31)	0.28 (0.19,0.37)	0.82 (0.8,0.85)	0.01 (0,0.02)	0.07 (-0.04,0.16)
0.12	0.48 (0.38,0.57)	0.77 (0.74,0.79)	0.02 (0,0.03)	0.17 (0.04,0.27)	0.22 (0.14,0.28)	0.88 (0.85,0.9)	0 (0,0.01)	0.05 (-0.05,0.13)
0.13	0.4 (0.32,0.5)	0.82 (0.79,0.84)	0.01 (0,0.02)	0.13 (0.03,0.24)	0.19 (0.11,0.25)	0.93 (0.91,0.94)	0.01 (0,0.01)	0.08 (0,0.16)
0.14	0.34 (0.26,0.43)	0.85 (0.83,0.87)	0.01 (0,0.02)	0.1 (-0.01,0.21)	0.17 (0.1,0.23)	0.95 (0.93,0.96)	0.01 (0,0.01)	0.08 (0.01,0.15)
0.15	0.29 (0.2,0.38)	0.89 (0.87,0.91)	0.01 (0,0.02)	0.1 (0.01,0.21)	0.12 (0.06,0.17)	0.97 (0.96,0.98)	0.01 (0,0.01)	0.06 (0,0.12)
0.16	0.24 (0.17,0.31)	0.91 (0.89,0.92)	0.01 (0,0.01)	0.06 (-0.02,0.14)	0.07 (0.03,0.11)	0.98 (0.97,0.99)	0 (0,0.01)	0.03 (-0.03,0.07)
0.17	0.2 (0.13,0.26)	0.92 (0.91,0.94)	0 (0,0.01)	0.04 (-0.04,0.11)	0.06 (0.01,0.1)	0.99 (0.98,0.99)	0 (0,0.01)	0.03 (-0.01,0.07)
0.18	0.17 (0.1,0.23)	0.94 (0.93,0.96)	0 (0,0.01)	0.04 (-0.03,0.11)	0.03 (0,0.06)	0.99 (0.98,1)	0 (0,0)	0.01 (-0.02,0.04)
0.19	0.14 (0.07,0.2)	0.96 (0.94,0.97)	0 (0,0.01)	0.04 (-0.04,0.11)	0.02 (0,0.05)	1 (0.99,1)	0 (0,0)	0.01 (-0.01,0.04)
0.2	0.13 (0.07,0.19)	0.97 (0.96,0.98)	0 (0,0.01)	0.04 (-0.02,0.11)	0.02 (0,0.05)	1 (0.99,1)	0 (0,0)	0.01 (-0.01,0.04)
0.21	0.1 (0.04,0.15)	0.98 (0.97,0.98)	0 (0,0.01)	0.04 (-0.03,0.09)	0.01 (0,0.03)	1 (1,1)	0 (0,0)	0.01 (-0.01,0.03)
0.22	0.08 (0.03,0.13)	0.98 (0.98,0.99)	0 (0,0.01)	0.03 (-0.02,0.09)	0.01 (0,0.03)	1 (1,1)	0 (0,0)	0.01 (0,0.03)
0.23	0.07 (0.03,0.11)	0.99 (0.98,1)	0 (0,0.01)	0.04 (-0.02,0.09)	0.01 (0,0.03)	1 (1,1)	0 (0,0)	0.01 (0,0.03)
0.24	0.05 (0.02,0.09)	0.99 (0.99,1)	0 (0,0.01)	0.02 (-0.02,0.07)	0.01 (0,0.03)	1 (1,1)	0 (0,0)	0.01 (0,0.03)
0.25	0.04 (0.01,0.08)	0.99 (0.99,1)	0 (0,0.01)	0.02 (-0.03,0.05)	0.01 (0,0.03)	1 (1,1)	0 (0,0)	0.01 (0,0.03)

0.26	0.04 (0.01,0.08)	1 (0.99,1)	0 (0,0.01)	0.03 (-0.01,0.07)	0.01 (0,0.03)	1 (1,1)	0 (0,0)	0.01 (0,0.03)
0.27	0.04 (0.01,0.08)	1 (0.99,1)	0 (0,0.01)	0.03 (-0.01,0.07)	0.01 (0,0.03)	1 (1,1)	0 (0,0)	0.01 (0,0.03)
0.28	0.03 (0,0.07)	1 (0.99,1)	0 (0,0.01)	0.02 (-0.02,0.06)	0.01 (0,0.03)	1 (1,1)	0 (0,0)	0.01 (0,0.03)
0.29	0.02 (0,0.05)	1 (0.99,1)	0 (0,0)	0.01 (-0.02,0.04)	0 (0,0)	1 (1,1)	0 (0,0)	0 (0,0)
0.3	0.02 (0,0.05)	1 (1,1)	0 (0,0)	0.02 (-0.01,0.05)	0 (0,0)	1 (1,1)	0 (0,0)	0 (0,0)
0.31	0.02 (0,0.05)	1 (1,1)	0 (0,0)	0.02 (-0.01,0.05)	0 (0,0)	1 (1,1)	0 (0,0)	0 (0,0)
0.32	0.01 (0,0.04)	1 (1,1)	0 (0,0)	0.01 (-0.01,0.03)	0 (0,0)	1 (1,1)	0 (0,0)	0 (0,0)
0.33	0.01 (0,0.04)	1 (1,1)	0 (0,0)	0 (-0.01,0.03)	0 (0,0)	1 (1,1)	0 (0,0)	0 (0,0)
0.34	0.01 (0,0.04)	1 (1,1)	0 (0,0)	0 (-0.02,0.03)	0 (0,0)	1 (1,1)	0 (0,0)	0 (0,0)
0.35	0.01 (0,0.04)	1 (1,1)	0 (0,0)	0.01 (0,0.04)	0 (0,0)	1 (1,1)	0 (0,0)	0 (0,0)
0.36	0.01 (0,0.04)	1 (1,1)	0 (0,0)	0.01 (0,0.04)	0 (0,0)	1 (1,1)	0 (0,0)	0 (0,0)
0.37	0.01 (0,0.04)	1 (1,1)	0 (0,0)	0.01 (0,0.04)	0 (0,0)	1 (1,1)	0 (0,0)	0 (0,0)
0.38	0.01 (0,0.04)	1 (1,1)	0 (0,0)	0.01 (0,0.04)	0 (0,0)	1 (1,1)	0 (0,0)	0 (0,0)
0.39	0.01 (0,0.04)	1 (1,1)	0 (0,0)	0.01 (0,0.04)	0 (0,0)	1 (1,1)	0 (0,0)	0 (0,0)
0.4	0.01 (0,0.04)	1 (1,1)	0 (0,0)	0.01 (0,0.04)	0 (0,0)	1 (1,1)	0 (0,0)	0 (0,0)
0.41	0.01 (0,0.04)	1 (1,1)	0 (0,0)	0.01 (0,0.04)	0 (0,0)	1 (1,1)	0 (0,0)	0 (0,0)
0.42	0.01 (0,0.04)	1 (1,1)	0 (0,0)	0.01 (0,0.04)	0 (0,0)	1 (1,1)	0 (0,0)	0 (0,0)
0.43	0 (0,0)	1 (1,1)	0 (0,0)	0 (0,0)	0 (0,0)	1 (1,1)	0 (0,0)	0 (0,0)
0.44	0 (0,0)	1 (1,1)	0 (0,0)	0 (0,0)	0 (0,0)	1 (1,1)	0 (0,0)	0 (0,0)
0.45	0 (0,0)	1 (1,1)	0 (0,0)	0 (0,0)	0 (0,0)	1 (1,1)	0 (0,0)	0 (0,0)
0.46	0 (0,0)	1 (1,1)	0 (0,0)	0 (0,0)	0 (0,0)	1 (1,1)	0 (0,0)	0 (0,0)
0.47	0 (0,0)	1 (1,1)	0 (0,0)	0 (0,0)	0 (0,0)	1 (1,1)	0 (0,0)	0 (0,0)
0.48	0 (0,0)	1 (1,1)	0 (0,0)	0 (0,0)	0 (0,0)	1 (1,1)	0 (0,0)	0 (0,0)
0.49	0 (0,0)	1 (1,1)	0 (0,0)	0 (0,0)	0 (0,0)	1 (1,1)	0 (0,0)	0 (0,0)
**0.5**	**0 (0,0)**	**1 (1,1)**	**0 (0,0)**	**0 (0,0)**	0 (0,0)	1 (1,1)	0 (0,0)	0 (0,0)

Supplementary Table 5: Model comparisons including coefficients for development and internal validation

Model	Model Predictors of TRS	Coefficients after shrinkage for optimism[$]	Shrinkage factor	Pooled Development Sample performance statistics	Internal validation. Distribution of predicted probabilities	Internal validation. Calibration plot
M1	*Intercept*<br>Sex	*-2.9143866*<br>0.5836307	1	C: 0.56 (0.50-0.62)<br>Brier score: 0.07		
M2	*Intercept*<br>Sex<br>Age<br>Black/Afro-Caribbean ethnicity<br>Asian ethnicity	*-1.853393*<br>0.40447791<br>-0.03774630<br>0.48783825<br>-0.00957477	0.85	C: 0.64 (0.57-0.71)<br>Brier score: 0.07		
M3	*Intercept*<br>Sex<br>Age<br>Black/Afro-Caribbean ethnicity<br>Asian ethnicity<br>Triglycerides	*-1.980062*<br>0.31138621<br>-0.04040389<br>0.43925284<br>-0.08269676<br>0.18532220	0.82	C: 0.68 (0.61-0.75)<br>Brier score: 0.07	Supplementary Figure 7	Supplementary Figure 8
M4	*Intercept*<br>Sex<br>Age<br>Black/Afro-Caribbean ethnicity<br>Asian ethnicity<br>Triglycerides<br>Alkaline phosphatase (ALP)	*-2.619038*<br>0.280463977<br>-0.037394517<br>0.456980896<br>-0.104559485<br>0.165891049<br>0.007446233	0.81	C: 0.69 (0.62-0.76)<br>Brier score: 0.07	Supplementary Figure 9	Supplementary Figure 10
Main Forced entry	*Intercept*<br>Sex<br>Age<br>Black/Afro-Caribbean ethnicity<br>Asian ethnicity<br>Triglycerides<br>Alkaline phosphatase (ALP)<br>Lymphocyte count	*-2.827381*<br>0.286466741<br>-0.036205346<br>0.419614174<br>-0.144147329<br>0.149214138<br>0.006713513<br>0.131215526	0.79	C: 0.70 (0.63-0.76)<br>Brier score: 0.07	Supplementary Figure 3	Supplementary Figure 5
Main LASSO	*Intercept*<br>Sex<br>Age<br>Black/Afro-Caribbean ethnicity<br>Asian ethnicity<br>Triglycerides<br>Alkaline phosphatase (ALP)<br>Lymphocyte count<br>Smoking status<br>Body mass index (BMI)<br>Random glucose<br>Neutrophil count	*-2.736365*<br>0.132050<br>-0.248397<br>0.304147<br>-0.002375<br>0.139795<br>0.131153<br>0.060623<br>0.057593<br>-0.026467<br>-0.027369<br>-0.012826	N/A	C: 0.69 (0.63-0.77)<br>Brier score: 0.07	Supplementary Figure 4	Supplementary Figure 6

C, C value (95% confidence interval) (see Methods).
[$]The coefficients are relative to non-scaled values for forced-entry models, and to scaled and centered values for the LASSO model.

Supplementary Figures

Supplementary Figure 1: PubMed results for "risk AND prediction AND psychosis" by year

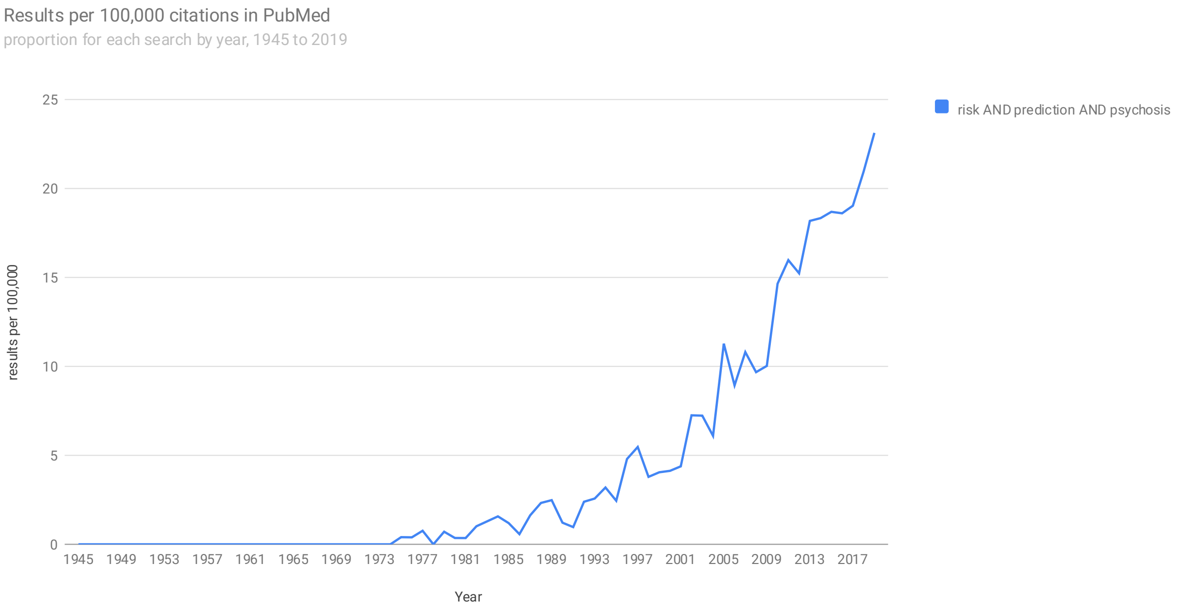

Generated with Sperr E. PubMed by Year [Internet]. 2016 [cited 2022]. Available from http://esperr.github.io/pubmed-by-year/

Supplementary Figure 2: Internal validation. Distribution of predicted probabilities for the main model – forced entry

X axis: predicted probability

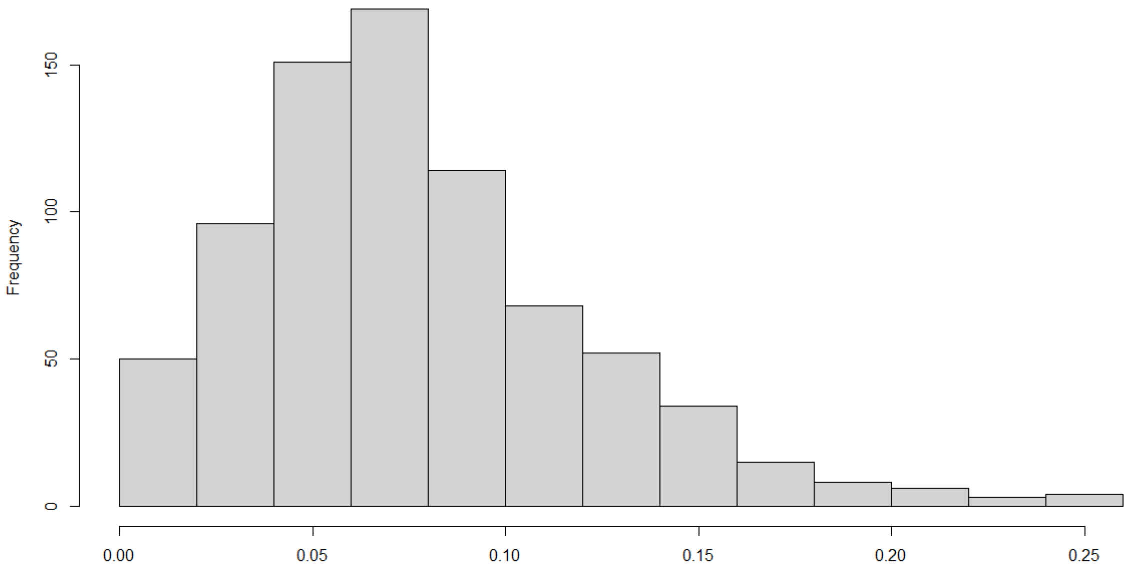

Supplementary Figure 3: Internal validation. Distribution of predicted probabilities for the main model – LASSO

X axis: predicted probability

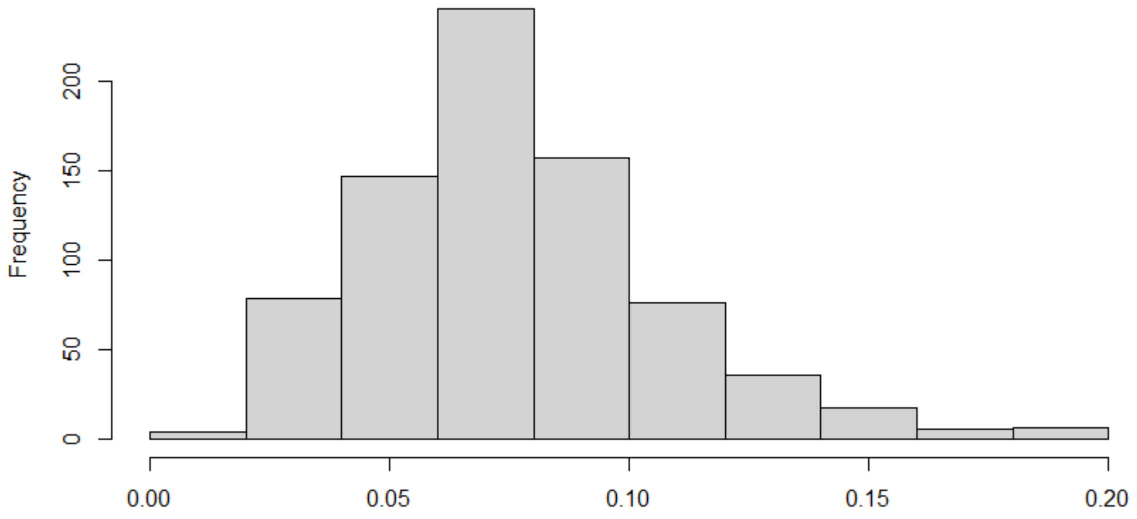

Supplementary Figure 4: Internal validation. Calibration plot (expected vs predicted probabilities) for the main model – forced entry

Model calibration is the extent to which outcomes predicted by the model are similar to those observed in the validation dataset.
Calibration plots illustrate agreement between observed risk (y axis) and predicted risk (x axis). Perfect agreement would trace the red line. Model calibration is shown by the continuous black line. Triangles denote grouped observations for participants at deciles of predicted risk, with 95% CIs indicated by the vertical black lines. Axes range between 0 and 0.3 since very few individuals received predicted probabilities greater than 0.3.

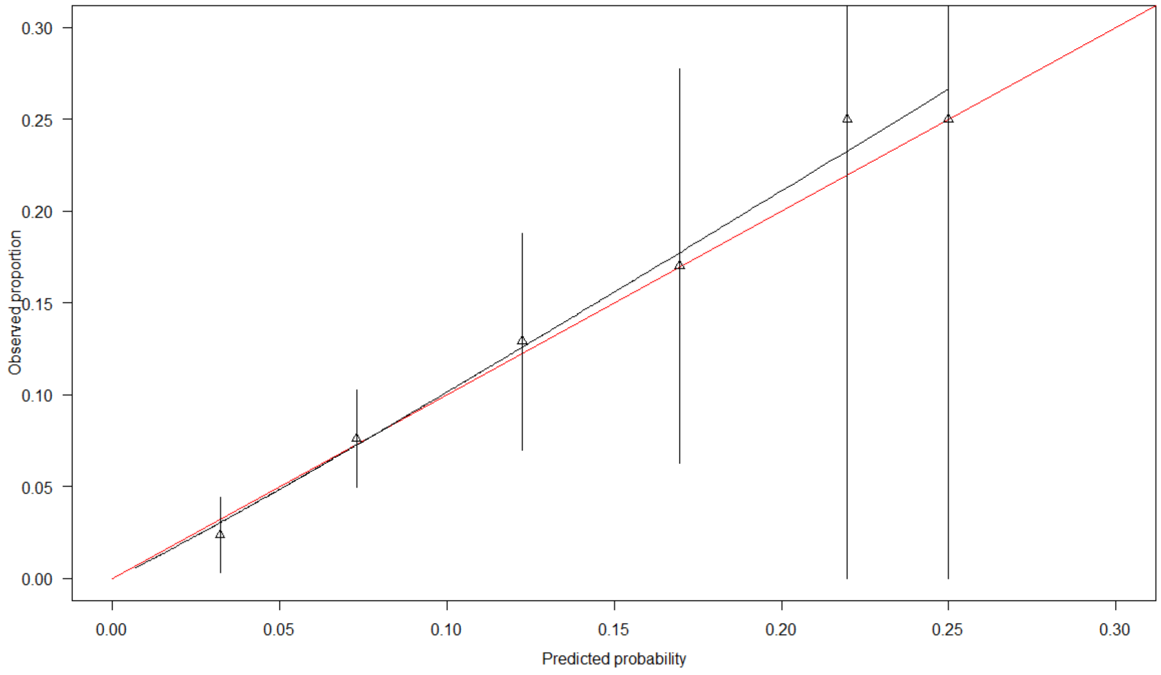

Supplementary Figure 5: Internal validation. Calibration plot (expected vs predicted probabilities) for the main model – LASSO

Model calibration is the extent to which outcomes predicted by the model are similar to those observed in the validation dataset.
Calibration plots illustrate agreement between observed risk (y axis) and predicted risk (x axis). Perfect agreement would trace the red line. Model calibration is shown by the continuous black line. Triangles denote grouped observations for participants at deciles of predicted risk, with 95% CIs indicated by the vertical black lines. Axes range between 0 and 0.3 since very few individuals received predicted probabilities greater than 0.3.

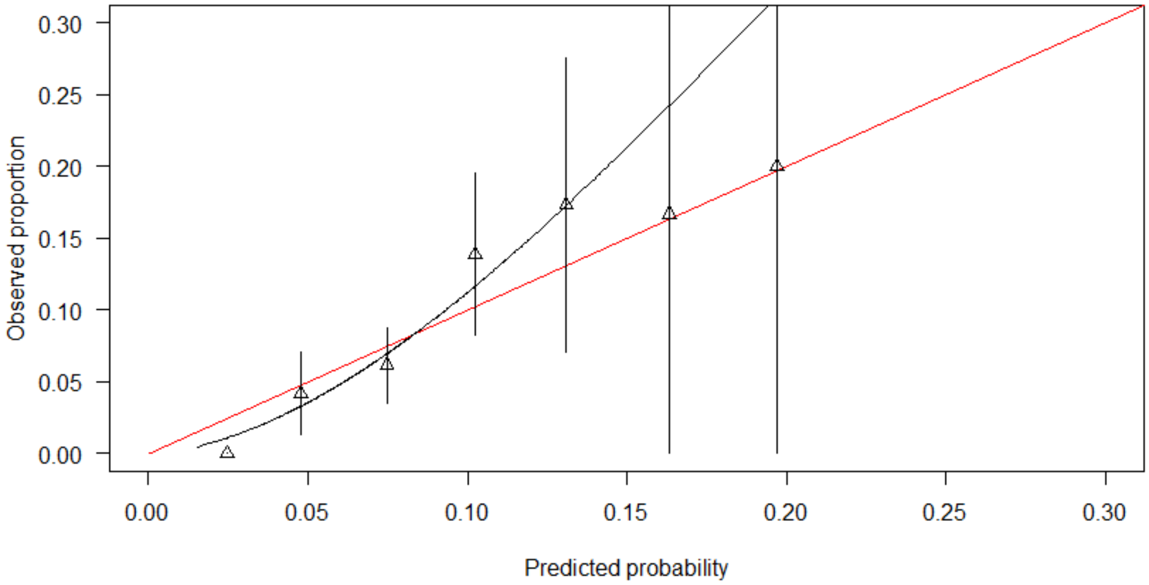

Supplementary Figure 6: Internal validation. Distribution of predicted probabilities for M3

X axis: predicted probability

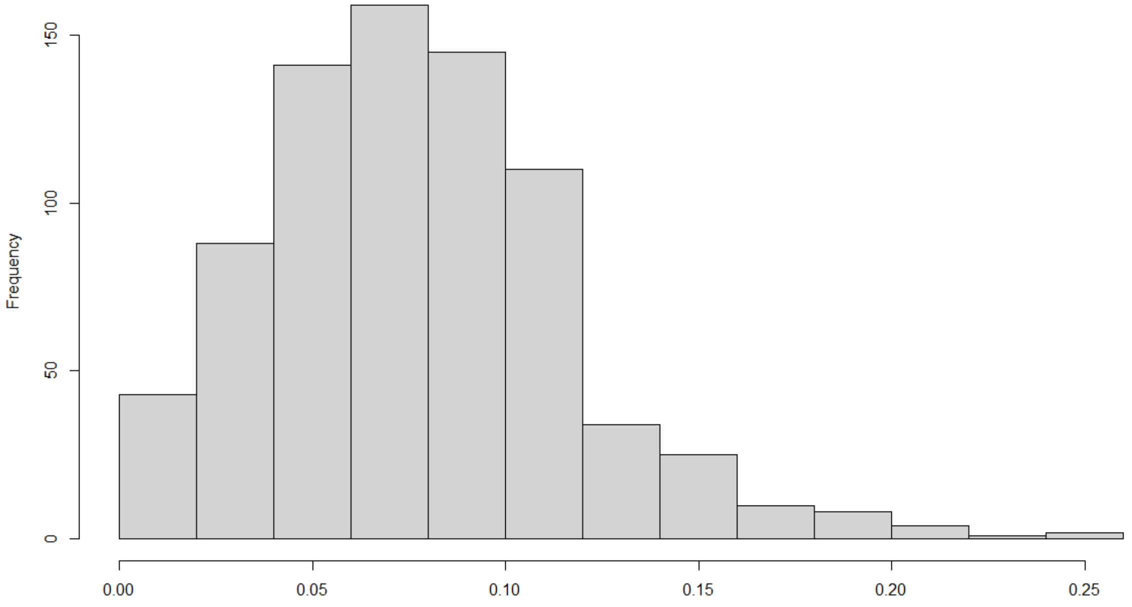

Supplementary Figure 7: Internal validation. Calibration plot (expected vs predicted probabilities) for M3

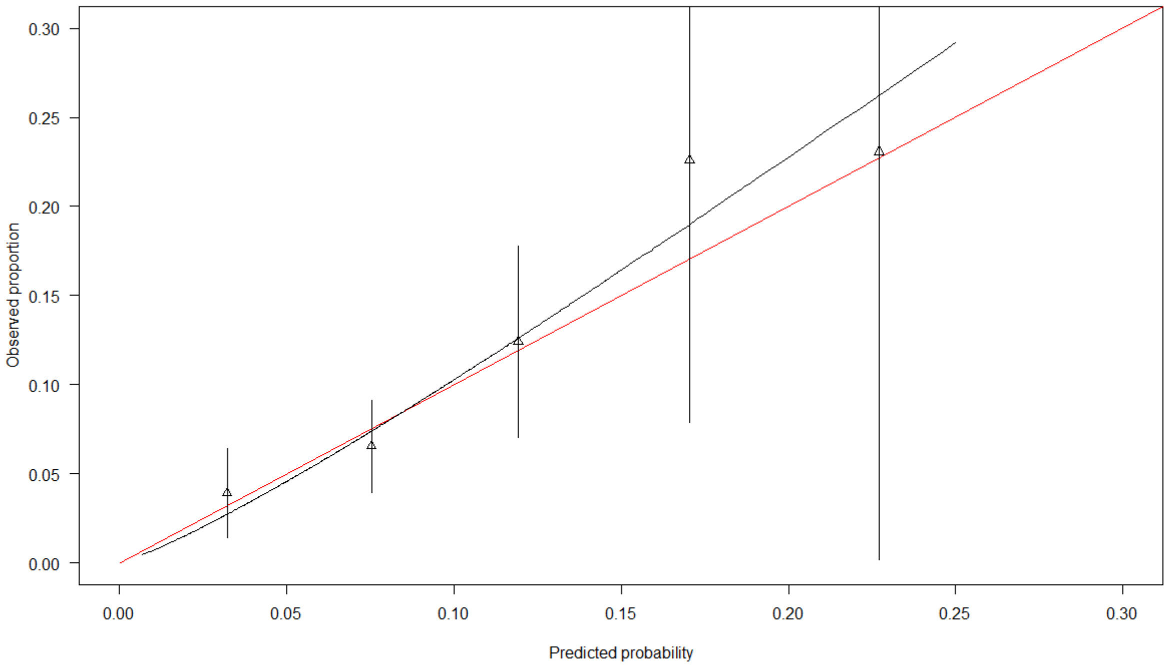

Supplementary Figure 8: Internal validation. Distribution of predicted probabilities for M4

X axis: predicted probability

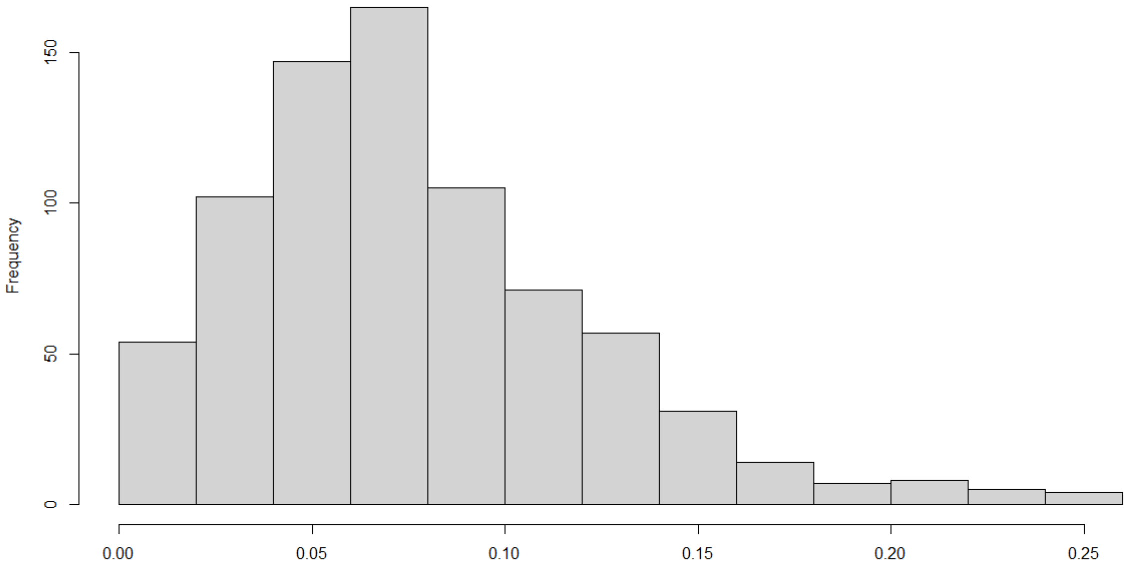

Supplementary Figure 9: Internal validation. Calibration plot (expected vs predicted probabilities) for M4

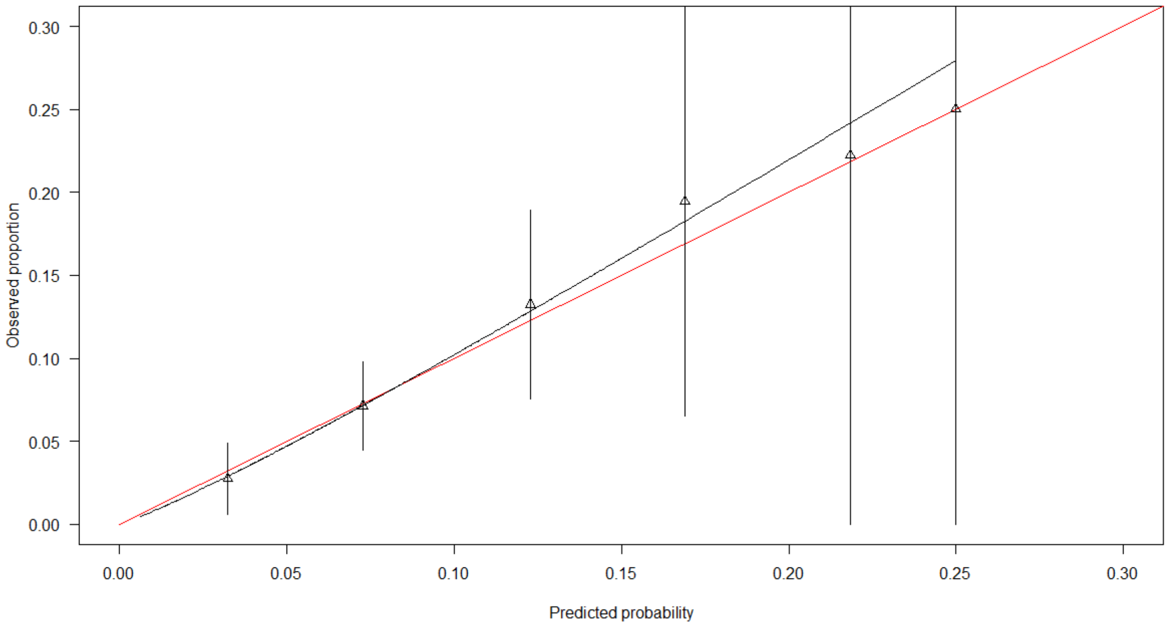

Supplementary References

1. Cardinal RN. Clinical records anonymisation and text extraction (CRATE): an open-source software system. *BMC Medical Informatics and Decision Making* 2017; **17**(1)**:** 50.

2. Riley RD, Snell KI, Ensor J, Burke DL, Harrell Jr FE, Moons KG *et al.* Minimum sample size for developing a multivariable prediction model: PART II-binary and time-to-event outcomes. *Statistics in medicine* 2019; **38**(7)**:** 1276-1296.

3. Ensor J, Martin EC, Riley RD. pmsampsize: Calculates the Minimum Sample Size Required for Developing a Multivariable Prediction Model. 2021.

4. R Core Team. R: A language and environment for statistical computing [Software]. R Foundation for Statistical Computing: Vienna, Austria, 2021.

5. Lally J, Ajnakina O, Di Forti M, Trotta A, Demjaha A, Kolliakou A *et al.* Two distinct patterns of treatment resistance: clinical predictors of treatment resistance in first-episode schizophrenia spectrum psychoses. *Psychological medicine* 2016; **46**(15)**:** 3231-3240.

6. Meltzer HY. Treatment-resistant schizophrenia-the role of clozapine. *Current medical research and opinion* 1997; **14**(1)**:** 1-20.

7. Siskind D, Orr S, Sinha S, Yu O, Brijball B, Warren N *et al.* Rates of treatment-resistant schizophrenia from first-episode cohorts: systematic review and meta-analysis. *The British Journal of Psychiatry* 2021**:** 1-6.

8. Demjaha A, Lappin J, Stahl D, Patel M, MacCabe J, Howes O *et al.* Antipsychotic treatment resistance in first-episode psychosis: prevalence, subtypes and predictors. *Psychological medicine* 2017; **47**(11)**:** 1981-1989.

9. Van Buuren S, Groothuis-Oudshoorn K. mice: Multivariate imputation by chained equations in R. *Journal of statistical software* 2011; **45:** 1-67.

10. Multiple imputation with large proportions of missing data: How much is too much? *Proceedings of the United Kingdom Stata Users' Group Meetings 2011*2011. Stata Users Group.

11. Dong Y, Peng C-YJ. Principled missing data methods for researchers. *SpringerPlus* 2013; **2**(1)**:** 1-17.

12. Madley-Dowd P, Hughes R, Tilling K, Heron J. The proportion of missing data should not be used to guide decisions on multiple imputation. *Journal of clinical epidemiology* 2019; **110:** 63-73.

13. Osimo EF, Perry BI, Cardinal RN, Lynall ME, Lewis J, Kudchadkar A *et al.* Inflammatory and cardiometabolic markers at presentation with first episode psychosis and long-term clinical

outcomes: A longitudinal study using electronic health records. *Brain Behav Immun* 2021; **91:** 117-127.

14. Osimo EF, Cardinal RN, Jones PB, Khandaker GM. Prevalence and correlates of low-grade systemic inflammation in adult psychiatric inpatients: An electronic health record-based study. *Psychoneuroendocrinology* 2018; **91:** 226-234.

15. Smart S, Kępińska A, Murray R, MacCabe J. Predictors of treatment resistant schizophrenia: a systematic review of prospective observational studies. *Psychological medicine* 2021; **51**(1)**:** 44-53.

16. Lever J, Krzywinski M, Altman N. Points of significance: model selection and overfitting. *Nature methods* 2016; **13**(9)**:** 703-705.

17. Spauwen J, Krabbendam L, Lieb R, Wittchen H-U, van Os J. Sex differences in psychosis: normal or pathological? *Schizophrenia research* 2003; **62**(1-2)**:** 45-49.

18. Perry BI, Upthegrove R, Crawford O, Jang S, Lau E, McGill I *et al.* Cardiometabolic risk prediction algorithms for young people with psychosis: a systematic review and exploratory analysis. *Acta Psychiatrica Scandinavica* 2020; **142**(3)**:** 215-232.

19. Perry BI, Osimo EF, Upthegrove R, Mallikarjun PK, Yorke J, Stochl J *et al.* Development and external validation of the Psychosis Metabolic Risk Calculator (PsyMetRiC): a cardiometabolic risk prediction algorithm for young people with psychosis. *The Lancet Psychiatry* 2021.

20. Nettis MA, Pergola G, Kolliakou A, O'Connor J, Bonaccorso S, David A *et al.* Metabolic-inflammatory status as predictor of clinical outcome at 1-year follow-up in patients with first episode psychosis. *Psychoneuroendocrinology* 2019; **99:** 145-153.

21. Barreira TV, Harrington DM, Staiano AE, Heymsfield SB, Katzmarzyk PT. Body adiposity index, body mass index, and body fat in white and black adults. *Jama* 2011; **306**(8)**:** 828-830.

22. Ikejima K, Lang T, Yamashina S, Enomoto N, Takei Y, Sato N. Role of Leptin in Pathogenesis of NASH. *NASH and Nutritional Therapy*. Springer2005, pp 44-49.

23. Morlán-Coarasa MJ, Arias-Loste MT, de la Foz VO-G, Martínez-García O, Alonso-Martín C, Crespo J *et al.* Incidence of non-alcoholic fatty liver disease and metabolic dysfunction in first episode schizophrenia and related psychotic disorders: a 3-year prospective randomized interventional study. *Psychopharmacology* 2016; **233**(23)**:** 3947-3952.

24. Machado MV, Diehl AM. Pathogenesis of nonalcoholic steatohepatitis. *Gastroenterology* 2016; **150**(8)**:** 1769-1777.

25. Miller BJ, Gassama B, Sebastian D, Buckley P, Mellor A. Meta-analysis of lymphocytes in schizophrenia: clinical status and antipsychotic effects. *Biol Psychiatry* 2013; **73**(10)**:** 993-999.

26. Karageorgiou V, Milas GP, Michopoulos I. Neutrophil-to-lymphocyte ratio in schizophrenia: A systematic review and meta-analysis. *Schizophrenia research* 2019; **206:** 4-12.

27. Gurillo P, Jauhar S, Murray RM, MacCabe JH. Does tobacco use cause psychosis? Systematic review and meta-analysis. *The Lancet Psychiatry* 2015; **2**(8)**:** 718-725.

28. Perry BI, McIntosh G, Weich S, Singh S, Rees K. The association between first-episode psychosis and abnormal glycaemic control: systematic review and meta-analysis. *The Lancet Psychiatry* 2016; **3**(11)**:** 1049-1058.

29. Perry BI, Stochl J, Upthegrove R, Zammit S, Wareham N, Langenberg C *et al.* Longitudinal trends in childhood insulin levels and body mass index and associations with risks of psychosis and depression in young adults. *JAMA psychiatry* 2021; **78**(4)**:** 416-425.
